# Modeling of celiac disease immune response and the therapeutic effect of potential drugs

**DOI:** 10.1186/1752-0509-7-56

**Published:** 2013-07-05

**Authors:** Oleg O Demin, Sergey V Smirnov, Victor V Sokolov, Lourdes Cucurull-Sanchez, Cesar Pichardo-Almarza, M Victoria Flores, Neil Benson, Oleg V Demin

**Affiliations:** 1 Institute for System Biology SPb, Moscow, Russia; 2 Pfizer Global R&D, Sandwich, UK; 3 Present affiliation: GlaxoSmithKline Medicines Research Centre, Stevenage, UK; 4 Present affiliation: InScilico Ltd, London, UK; 5 Present affiliation: eTherapeutics plc, Long Hanborough, UK; 6 Present affiliation: Xenologiq Ltd, Canterbury, UK

**Keywords:** Celiac disease, Mathematical modeling, Gluten, Drug development, Immune response

## Abstract

**Background:**

Celiac disease (CD) is an autoimmune disorder that occurs in genetically predisposed people and is caused by a reaction to the gluten protein found in wheat, which leads to intestinal villous atrophy. Currently there is no drug for treatment of CD. The only known treatment is lifelong gluten-free diet. The main aim of this work is to develop a mathematical model of the immune response in CD patients and to predict the efficacy of a transglutaminase-2 (TG-2) inhibitor as a potential drug for treatment of CD.

**Results:**

A thorough analysis of the developed model provided the following results:

1. TG-2 inhibitor treatment leads to insignificant decrease in antibody levels, and hence remains higher than in healthy individuals.

2. TG-2 inhibitor treatment does not lead to any significant increase in villous area.

3. The model predicts that the most effective treatment of CD would be the use of gluten peptide analogs that antagonize the binding of immunogenic gluten peptides to APC. The model predicts that the treatment of CD by such gluten peptide analogs can lead to a decrease in antibody levels to those of normal healthy people, and to a significant increase in villous area.

**Conclusions:**

The developed mathematical model of immune response in CD allows prediction of the efficacy of TG-2 inhibitors and other possible drugs for the treatment of CD: their influence on the intestinal villous area and on the antibody levels. The model also allows to understand what processes in the immune response have the strongest influence on the efficacy of different drugs. This model could be applied in the pharmaceutical R&D arena for the design of drugs against autoimmune small intestine disorders and on the design of their corresponding clinical trials.

## Background

Celiac disease (CD) is an autoimmune disorder caused by the gluten protein contained in many grains. Upon entry of gluten into the small intestine, a patient develops painful digestion disorders due to villi impairment and loss of absorption. CD is partially a heritable disease [1]. The gene implicated in predisposition to CD (HLA DQ2 and DQ8) has a worldwide prevalence of around 1%. At present there is no therapeutic agent to treat this disorder. The only approach able to minimize the CD symptoms and currently used as prophylaxis is the strict compliance to a gluten-free diet (GFD), which consists on the removal of all gluten containing products, such as starchy and pasta from the diet, or the exchange of these products for products with a reduced gluten content [2]. Similar to many other autoimmune diseases, CD involves innate and adaptive immune responses [3,4]. The innate response causes a primary impairment of villi in the small intestine, increasing epithelial permeability allowing the entry of proteins from the lumen to the lamina propria [1]. The adaptive immune response involves the binding of peptides and/or proteins present in the intestinal lamina propia to antigen-presenting cells (APCs) which leads to antibody production [1]. These peptides can undergo deamidation by the enzyme tissue transglutaminase (TG-2) [1], which in genetically predisposed individuals can boost the peptide immunogenicity [5,6]. This deamidation process enables the APCs to take up not only immunogenic peptides but also TG-2-peptide complexes. As a consequence, antibodies are produced not only against gluten peptides but also against TG-2 [7-10]. The two markers of CD are a decline in the villous area of the small intestine (i.e. villous breakdown), and the appearance of anti TG-2 antibody in plasma. This suggests that TG-2 is a key component of the disease and therefore a potential target candidate for CD therapy. Nevertheless, there are many unexplored aspects in CD pathogenesis such as breakdown of oral tolerance and non DQ2/DQ8 ways of adaptive immune response stimulation.

To describe all intra- and extracellular processes associated with a disease and integrate heterogeneous sets of experimental data, a Quantitative Systems Pharmacology (QSP) modeling approach can be employed. This approach enables the description of a general mechanism of the immune response and its integration into the regulatory mechanisms characteristic of a disease of interest, in the current case CD. In addition, the QSP modeling approach provides a way for integrating the experimental data into a model to appropriately describe the disease of interest; with the aim of evaluating the most important processes and predicting potential targets for therapy.

A mathematical model of CD or its immune response has not yet been described in the literature. However, the mathematical modeling of a general immune response has been extensively performed and described, in particular the adaptive immune response, i.e. antigen presentation, synthesis, and activity of antibodies. The majority of the examples in the literature only describe general trends of the adaptive immune response to an antigen, without taking into account the complex regulatory mechanisms associated to the specific disease [11-14]. Just a limited number of publications focus on the adaptive immune response in the context of a disease mechanism in particular those induced by bacterial agents [15,16]. The work presented here intends to fill in these gaps by constructing a model of the innate and adaptive immune responses in CD. This model integrates all the human *in vitro*, *ex vivo* and *in vivo* data available, enabling the prediction of the efficacy of a TG-2 inhibitor, as well as the effect of other possible therapeutic agents on the levels of anti-TG-2 antibodies in plasma, and on the villous area in the small intestine.

## Methods

### Available experimental data, facts and assumptions used for model development

The model was constructed on the basis of the following experimental and literature information:

1) Healthy subjects do not have DQ2/DQ8 APCs [1].

2) Gluten peptides bind to receptors of intestinal epithelial cells (IEC), thus inducing zonulin synthesis that breaks down tight cell junctions [17,18].

3) CD patients have a high level of intraepithelial lymphocytes (IEL), including activated IELs [19,20].

4) Natural killers induce IEC apoptosis [21-23].

5) CD patients have an elevated level of interleukin-15 (IL-15) [24].

6) IL-15 promotes differentiation of APCs from monocytes, stimulates activation of IELs and arrests their apoptosis [24-26].

7) T helpers of type 1 and type 17 are the main types of T-cells in adaptive immune response [1,27-29].

8) CD patients have an elevated level of interferon γ (IFN-γ) in comparison to healthy individuals [30].

9) CD patients have an increased level of interleukin-21 (IL-21) relative to healthy individuals [31,32].

10) IFN-γ triggers IEC apoptosis [33].

11) IL-21 triggers IEC apoptosis [33].

12) IFN-γ and IL-21 are synthesized by activated Т-cells and activated IELs, i.e. natural killers [33-35].

13) CD patients test is positive for antibodies to gluten peptides and to TG-2 [10].

14) Antibodies to gluten peptides and TG-2 induce IEC apoptosis and inhibit their maturation [36].

15) CD patients have higher constitutive expression of IL15 receptor alpha in comparison with healthy subjects [37]. Binding of IL-15 to these receptors leads to IEL activation

16) The threshold of IEL activation by IL-15 is lower in CD patients than that in healthy subjects [37-39].

17) CD patients have higher zonulin level in comparison with healthy subjects [40,41].

In the development of this model the following assumptions were made:

a) T-helpers of types 1 and 17 are combined in one variable which is designated as T-cells.

a) Since the synthesis and degradation rates of IFN-γ and IL-21, as well as their action on IEC death are similar, IFN-γ and IL-21 were merged into a single variable named as IF-21. The IF-21 synthesis rate was defined as combination of IFN-γ and IL-21 synthesis velocities, and the IF-21 degradation rate was set to the average between IFN-γ and IL-21 degradation rates (see the section “Identification of model parameters” below).

a) There are no both innate (based on clauses (3), (5), (15)-(17)) and adaptive (based on clause (1)) immune responses in healthy subjects. In the model describing healthy subjects IEC activation, IEL activation velocities are equal to zero and there are no differential equations for APC with DQ2/DQ8 histocompatibility complex. As a result, level of all activated cells, cytokines, zonulin and antibodies are equal to zero for healthy subjects.

a) The permeability of intestinal wall depends on zonulin level and number of IEC. Velocity of gluten peptides transport through intestine wall is equal to zero when there is no zonulin and level of IEC corresponds to healthy subject level. The influence of zonulin and IEC is described in additive manner (for more details see Additional file 1).

a) To take into account a delay in antibody production caused by T cells activation [42] and affinity maturation of B cells [43], a transient step in T-cell activation was introduced.

a) The arrival of gluten peptides into the lumen of healthy subjects and celiac disease patients on gluten-containing diet was considered constant rather than discrete and at a rate of 3 times per day, which would correspond to a normal daily food intake. It was set to the average inflow in a patient following a GFD diet, which according to the Codex Draft Revise Standard (2000) should contain less that 20 ppm of gluten for foodstuffs naturally gluten-free (GF) and less than 200 ppm for foodstuffs rendered gluten-free (GF) [44].

### Scheme of the processes

**Figure 1:**
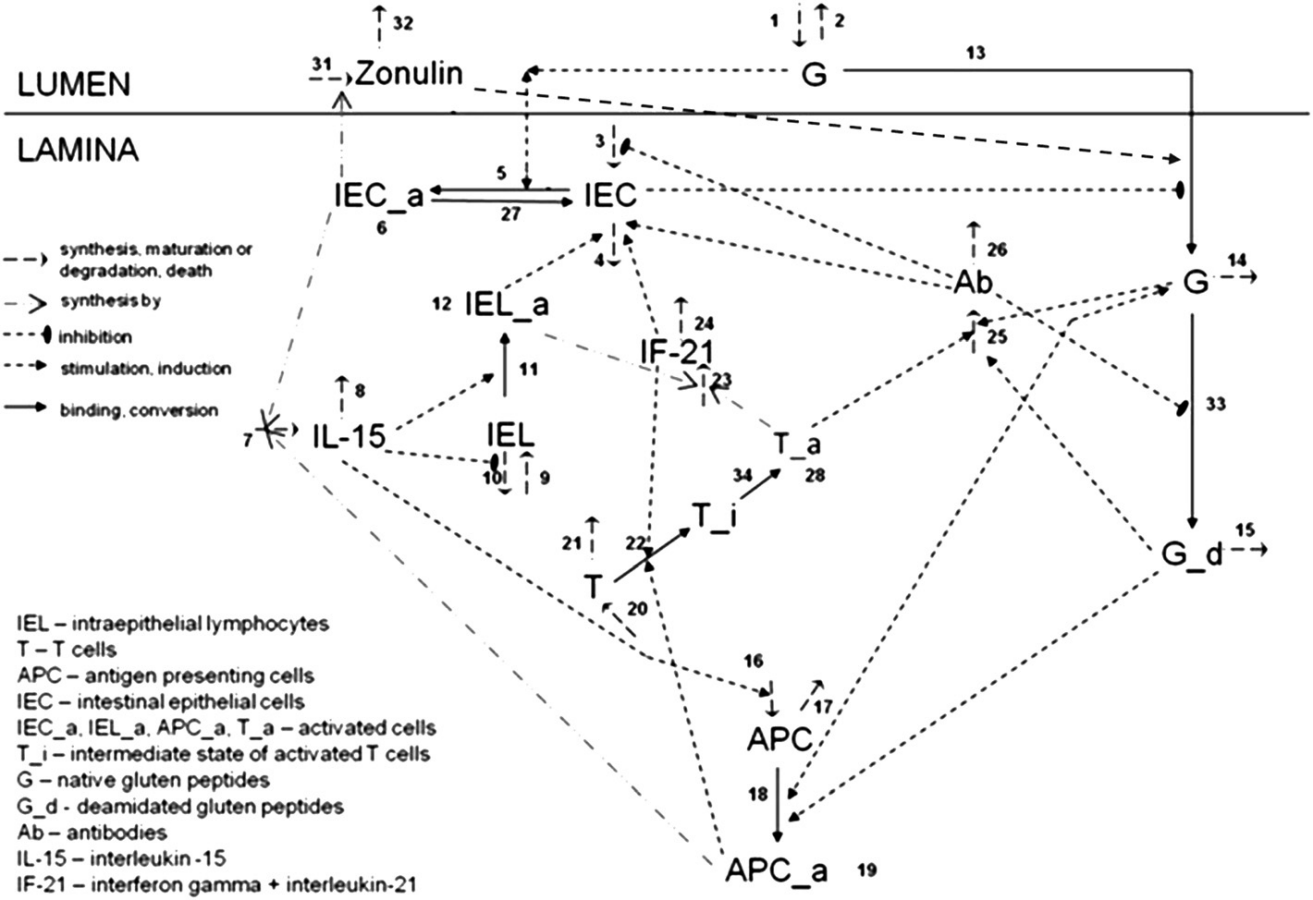
**Network of processes of innate and adaptive immune responses described in the model.** IEC activation (reaction #5), IEL activation (reaction #11) velocities are equal to zero and there are no differential equations for DQ2/DQ8 APC in the model for healthy subjects.

Based on the experimental data and assumptions stated above a network of processes was built, describing innate and adaptive immune responses in CD patients (Figure 1). The innate immunity cascade begins with the binding of gluten peptides to IEC receptors (reaction #5), thus activating them (see clause (2) in the previous section). These activated IECs produce zonulin and release it into the lumen (reaction #31) and IL-15 into the lamina (reaction #7) respectively. Zonulin promotes the disruption of tight junctions that control the permeability of macromolecules through the intestinal epithelial barrier (reaction #13) (see clause (2)). IL-15 activates the IELs and inhibits their apoptosis (reaction #10 and #11) (see clause (6)). In accordance with clause (4) of the previous section, the activated IELs (natural killers) induce IEC apoptosis (reactions #4 and #6) and release various toxic proteins (granzymes, perforins, etc.). In addition, activated IELs produce IFN-γ and IL-21 (reaction #23) that also trigger IEC apoptosis (reactions #4 and #6) (see clauses (10)-(12)). The disruption of the tight junctions and IEC death lead to the impairment of the epithelial barrier and an increase of its permeability, which allows the gluten peptides in the lumen to enter the lamina (reaction #13).

When the peptides reach the lamina propria they can undergo deamidation by TG-2 [1] (reaction #33). Deamidated peptides are more immunogenic than native peptides [5,6,45,46]. IL-15, synthesized by activated IECs, stimulates the conversion of dendritic cells and monocytes into APCs with the definite histocompatibility complex DQ2/DQ8 (reaction #16) (see clause (6)). These APCs bind both native and deamidated gluten peptides as well as deamidated gluten peptides complexed to TG-2 [1] (reaction #18). As a result, TG-2 also becomes an antigen. Deamidated gluten peptides are more potent in APC activation than native peptides [5,46]. Those APCs that have taken up the antigen become activated, starting the production of IL-15 (reaction #7) and stimulating T-cell activation (reaction #22 and #34) [33]. The activated T-cells synthesize IFN-γ and IL-21 (reaction #23) that in turn activate more T-cells (reaction #22), forming a positive feedback loop. In addition, those activated T-cells (Т-helpers types 1 and 17) considerably stimulate the production of antibodies specific to TG-2 and gluten peptides (reaction #25), which in turn trigger the apoptosis of IECs and inhibit their maturation (reaction #3, #4 and #6) [1,35,36].

### Model description

All processes of immune response are described as system of ordinary differential equations:

$$\frac{dx}{\mathrm{dt}}=N\cdot v,\;x\left(0\right)=x_{0}$$

Here, **x** = [x_1_,…,x_m_]^T^ is vector of model variables concentrations, **x**_**0**_ = [x_10_,…,x_m0_]^T^ is vector of initial concentrations of model variables, **v** = [v_1_,…,v_n_]^T^ is vector of reaction rates, **N** is stoichiometric matrix which has *n* columns and *m* rows. Detailed description of system of differential equations and reaction velocities are presented in Additional file 1.

Overall, the model has a total of 16 variables and 54 parameters. Variables are listed in Additional file 1: Table S1. Parameters, their values, and a description of the source from which the values were obtained are presented in section “Identification of model parameters” and listed in Additional file 1: Table S2.

### Identification of model parameters

As stated in the section “Scheme of the processes” the reaction rate expressions contain 54 parameters. A portion of these parameters was either taken directly from literature sources or estimated from literature data. Seventeen parameters were determined via the latter approach, amongst them the death constant of APCs, the degradation constant of gluten peptides, the dissociation constant of gliadin from IEC receptors, and the portion of receptors to be occupied by gluten peptides to activate IECs. Values of 2 parameters were assumed and 4 parameters were calculated using experimental data. These parameters and their corresponding values are given in Additional file 1: Table S2. The other 31 parameters were evaluated based on the best fitting to the appropriate experimental data, using the Hook-Jeeves method as implemented in the DBSolve Optimum package [47,48]. As a criterion of goodness of fit, the sum of squares error function was used: $f\left(k_{j,}K_{j}\right)={\sum_{i}^{n}\left(v_{i}-{\bar{v}}_{i}\right)}^{2}$

Where *n* is the total number of experimental points, $\bar{v_{i}}$ is the experimentally measured value of the variable or reaction rate, *v*_*i*_ is the corresponding value estimated from the model, *k*_*j*_, *K*_*j*_ are unknown parameters. To estimate the values of the unknown parameters, the error function (*f*) was minimized.

Given the large number of unknown parameters contained in the model, it was necessary to fit them against the maximum possible experimental data points and calculate the range of possible parameter values. Therefore data was extracted from *in vitro* and *ex vivo* studies, as well as clinical studies in patients at various stages of CD, and 95% confidential intervals were calculated for fitted parameters using method described in [49] (see Additional file 1: Table S2).

## Results

### Model validation

**Figure 2:**
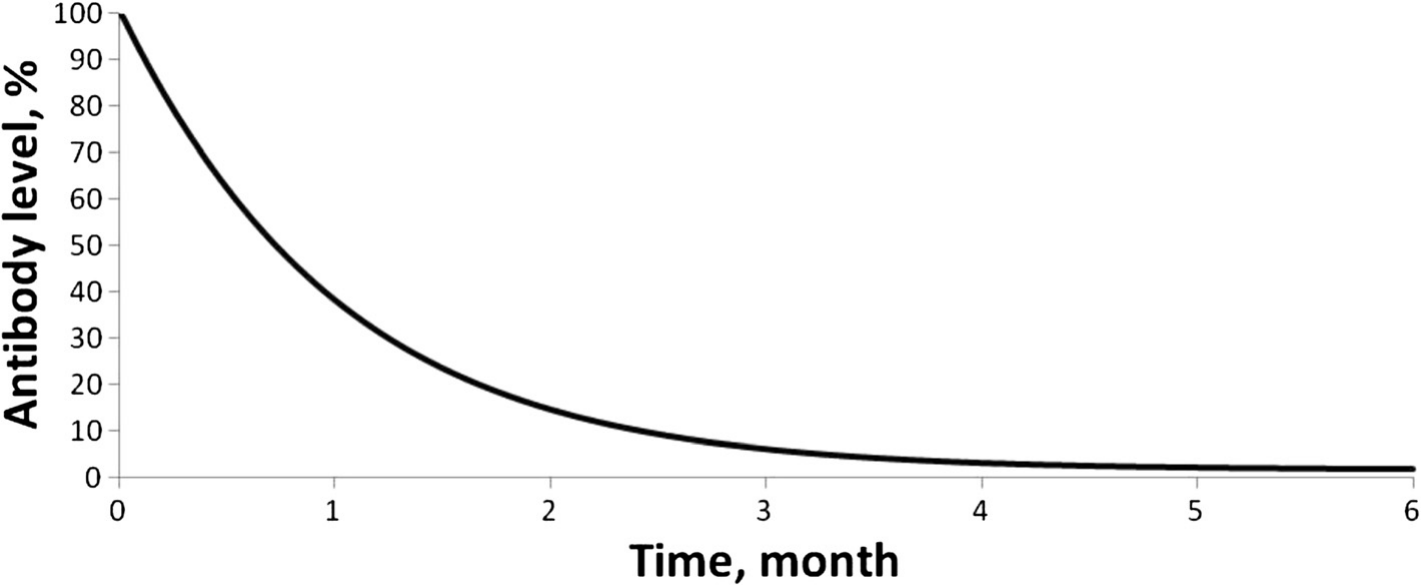
Decreasing of antibodies level after changing diet from gluten-containing to gluten-free.

**Figure 3:**
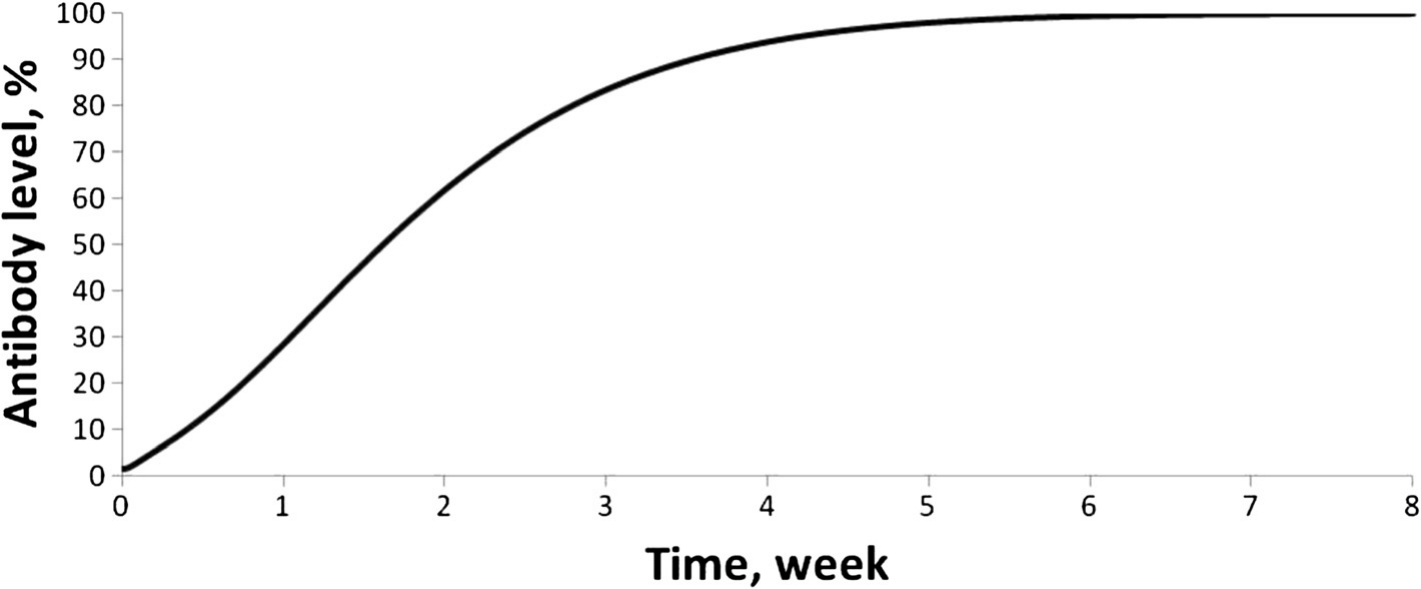
Appearance of antibodies after changing diet from gluten-free to gluten-containing.

The appropriate choice of model parameters was verified against independent data that had not been used in the identification of the model parameters. In fact, it is known that after changing over from a gluten-containing diet to a gluten-free diet the antibody level drops to the background level (in the model to zero level) within 2 to 4 months [50]. After changing over from a GFD to a gluten-containing diet, antibodies appear within 2 to 4 weeks [51]. Simulations of these two scenarios led to the following predictions: (i) after changing over from a gluten-containing diet to a GFD the antibody level drops nearly to zero within 2 to 3 months (Figure 2), (ii) after changing over from a GFD to a gluten-containing diet antibodies appear within 2 weeks (Figure 3). These temporal characteristics of transient processes, based on model calculations, are consistent with clinical data.

### Prediction of efficacy of various potential drugs to treat celiac disease

The model developed herein was used to predict the TG-2 inhibitor activity and other potential therapeutic agents for CD such as antibodies against IFN-γ and IL-15, gluten peptide-related agents that arrest activation of APCs (DQ2 blocking peptide analogues) [52-55], and gluten peptide-related agents that repress IEC activation (permeability inhibitors) [56]. The efficacy of each therapy was measured in terms of antibody levels and villous area levels. The antibody level was measured as a percentage, whereby 100% corresponds to the steady-state level of a patient on a gluten-containing diet. For a patient on a GFD and for a healthy patient the antibody levels are close to and equal to zero, respectively.

#### TG-2 inhibitor

**Figure 4:**
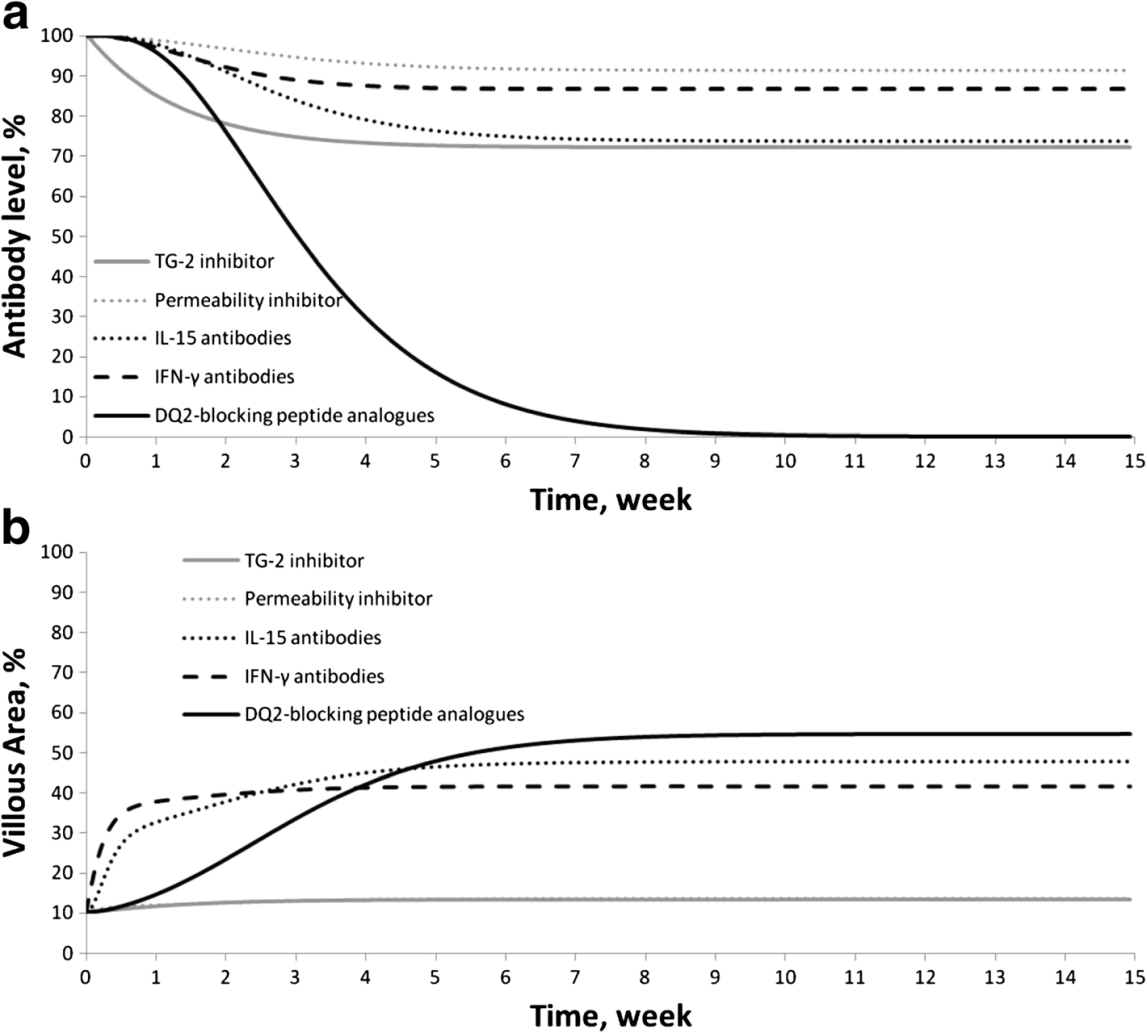
(a) Decreasing of antibodies level during treatment by different potential therapeutic agents; (b) Increasing of VA during treatment by different potential therapeutic agents (Curve for Permeability inhibitor coincides with curve for TG2 inhibitor).

To model the activity of a TG-2 inhibitor, the TG-2 level in the lamina propria was decreased. It was found that when the TG-2 level reaches zero, i.е. upon complete inhibition, the antibody levels only decrease to 70-75% (Figure 4a). Likewise, the complete inhibition of TG-2 does not cause a significant increase in the villous area: from 10 to 14% (Figure 4b).

**Figure 5:**
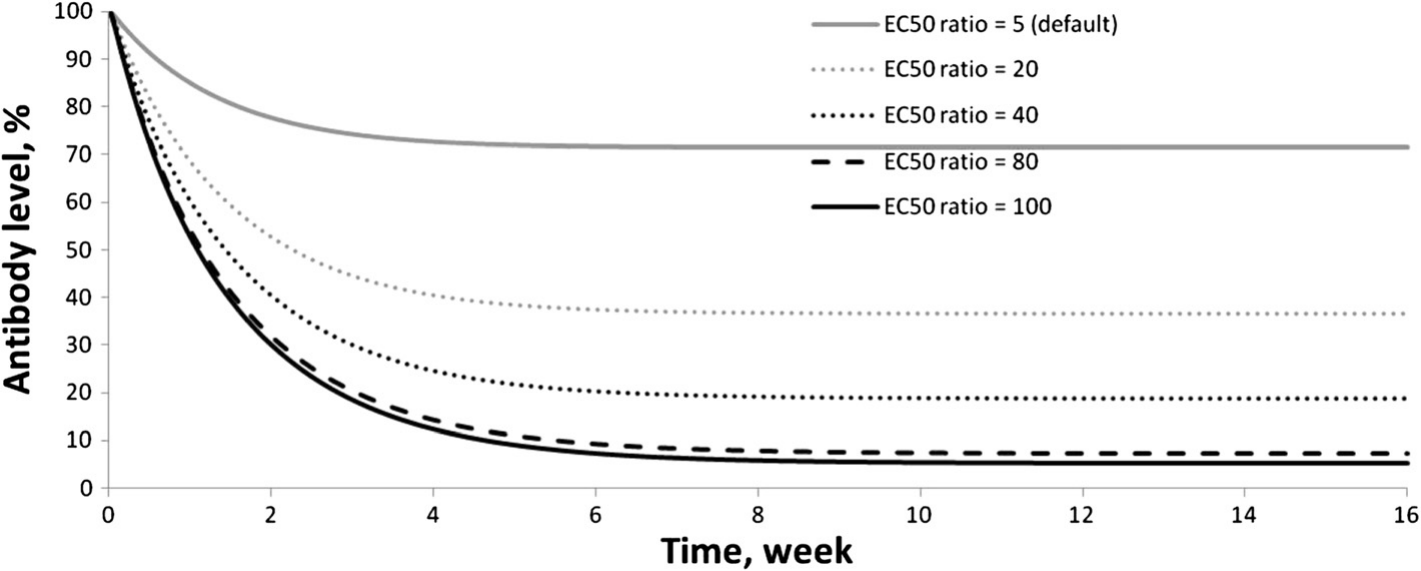
Decreasing of antibodies level during TG-2 inhibitor treatment at different EC50 ratio.

Next, an analysis was performed to determine what processes most significantly influence the efficacy of TG-2 inhibitors. It turned out that the most important process was the activation of APCs and B-cells by peptides binding to it. It is known from the literature that the ЕС50 of APC activation by deamidated peptides is 5-fold lower than by native peptides, i.e. deamidated proteins are more immunogenic [5,6]. However, these data cannot be considered unambiguous since the ratio of ЕС50 between deamidated and native forms varies with different gluten peptides [6]. So a series of simulations was run where the ЕС50 of the deamidated peptides was fixed and the EC50 of the native peptides was expressed as an increasing n-fold value of it. Every time the native to deamidated ЕС50 ratio was increased, the model was re-validated ($k_{s}^{\mathrm{ab}}$ was increased) since the antibody level declined due to a reduction in the immunogenicity of the native peptides. Overall, by increasing the native to deamidated ЕС50 ratio, the role of TG-2 in the immunogenicity of peptides becomes more important. The results shown that the antibody levels drop to or near zero at ratio values of 80-fold or greater (Figure 5), and no significant expansion of the villous area was observed.

#### IFN-γ antibodies

To model the activity of IFN-γ antibodies, the degradation rate constant of IF-21 (IFN-γ + IL-21) was increased. As a result, considering the maximum possible breakdown rate (i.e. when the IF-21 concentration decreased to zero) the antibody levels dropped to 83% (Figure 4a) and the villous area increased up to 40% (Figure 4b).

#### IL-15 antibodies

To model the activity of IL-15 antibodies the degradation rate constant of IL-15 was increased. At the maximum possible degradation rate (i.e. when IL-15 concentration decreased to zero) the antibody levels dropped to 69% (Figure 4a) and the villous area increased up to 46% (Figure 4b).

#### Gluten peptide-related agents that repress IEC activation (permeability inhibitors)

To model the activity of gluten peptides analogues the activation rate constant of IECs was set to zero. Consequently the antibody levels dropped to 92% (Figure 4a) and the villous area increased up to 14% (Figure 4b).

#### Gluten peptide-related agents that arrest activation of APCs (DQ2-blocking peptide analogues)

To model the activity of these analogues of gluten peptides the activation rate constant of APCs was set to zero. Consequently, the antibody levels dropped to zero (Figure 4a) and the villous area increased up to 51% (Figure 4b).

## Discussion

CD is a complex autoimmune disorder with many unexplored aspects. Role and contribution of various possible mechanisms (disruption of “central”, “peripheral” tolerances, T cell anergy, etc.) to oral tolerance breakdown, CD pathogenesis and severity is not established yet. There are different possible ways of gluten peptides transfer into the lamina propria [57]. They could cross the intestinal epithelium either via the paracellular route or via the transcellular route. In the first case this occurs as a consequence of an increased permeability, in the second case it occurs by using enterocytic vesicles that are able to cross the basal membrane allowing intact gluten peptides to have access to the lamina propria. It is known that one of the main cause of CD is the presence of HLA DQ2 and DQ8 molecules, but 1-2% of CD patients do not have DQ2 and/or DQ8 [51]. So there could be non DQ2/DQ8 ways of immune response stimulation. In our model the mechanism of oral tolerance breakdown has not been considered. We take into account only DQ2/DQ8 route of immune response stimulation and paracellular way of gluten peptides transfer into lamina propria. To describe healthy patients we use the assumption that there are no innate and adaptive immune responses in healthy subject (see section “Methods”). This approach allows us to simplify the equations of our model and to decrease the number of model parameters. The detailed description of mechanisms of IEL activation, transport of gluten peptides through intestine wall, interaction of peptides with non DQ2/DQ8 APC in healthy subjects could help to study and understand more clear the processes underlying the absence of immune responses in healthy subjects. This could be the next step for modeling of immune response in human.

The model described in this manuscript was able to predict that a TG-2 inhibitor can reduce the antibody levels down to zero if the deamidated peptides are more immunogenic than the native peptides, i.e. if the ЕС50 of deamidated peptides binding to APCs and B-cells is 80-fold lower or less than for native peptides. However, a TG-2 inhibitor would not significantly increase the villous area because the influence of antibodies on IEC death and inhibition of their maturation is insignificant. This is logical given that the villous area in a patient on a GFD was experimentally shown to be 50% of that in a healthy patient. It is known that in a patient following GFD the antibody levels are similar to the background antibody levels of a healthy patient (i.e. it equals to zero in the model). It follows from this that a 2-fold reduction in the villous area (from 100% to 50%) is due to IELs and IFN-γ, to a lesser extent, zonulin. After changing over from GFD to gluten-containing diet, the villous area decreases from 50% to 10%. This can be attributed to an increase in the levels of IELs and IFN-γ that trigger IEC apoptosis, as well as to the action of IL-21 and the antibodies. Overall, even if one suggests that the reduction from 50% to 10% only occurs through antibodies, then upon the condition that deamidated peptides are significantly more immunogenic than native ones (the ЕС50 ratio is 1:80 or greater), the TG-2 inhibitor induces a state similar to that of a GFD (in terms of both the antibody level and the villous area).

An evaluation of the efficacy of various potential drugs to treat CD suggests that the most efficient therapeutic agents would be analogues of immunogenic peptides that repress activation of APCs. This is logical as activated APCs play a key role in the adaptive immune response and maintenance of the activity of effectors of the innate immune response. Activated APCs release IL-15 that in turn arrests IELs death and at the same time induce their activation, i.e. stimulates differentiation of natural killers from IELs triggering apoptosis. As a conclusion, by arresting APC activation it seems that the synthesis of antibodies can be completely shut down and the concentrations of activated IELs, IFN-γ, and IL-21 significantly decreased, resulting in an increase of the villous area up to 51%.

One type of promising agents for CD therapy, the analogues of immunogenic peptides that repress IEC activation, appeared to be less efficient than GFD. These analogues arrest IEC activation, which represses the synthesis of zonulin and IL-15. However, this has no significant effect on effectors of the adaptive immune response. Activated APCs continue the synthesis of IL-15, activating IELs and T-cells, both of which trigger the release of IFN-γ and IL-21. The antibody level, which depend directly and exclusively on the number of activated T-cells and the amount of gluten peptides in the lamina propria, remain nearly the same as in the absence of peptide analogues. The villous area, increases only up to 14% due to the absence of zonulin, and a limited reduction in the concentration of activated IELs, IFN-γ and IL-21.

Significant reduction in IFN-γ and IL-15 level (resulted from administration of corresponding specific antibodies, for example) leads to the small decrease in antibody level. This can be explained in terms of auxiliary role of the cytokines in adaptive immune response. Indeed, IFN-γ is involved in T cells activation and elevation of IFN-γ results in increase in rate of T cells activation by some extent but even complete depletion of IFN-γ does not lead to stop in T cell activation. IL-15 is involved in differentiation of APCs from monocytes. Elevation in IL-15 is able to increase the rate of differentiation of APCs from monocytes but there is other IL-15 independent ways of replenishment of APC population. At the same time, decrease in IFN-γ and IL-15 levels results in significant increase of villous area (up to 40% and 46% respectively) because of the significant influence of IFN-γ and IL-21 on IEC apoptosis and strong stimulation effect of IL-15 on IEL activation.

The robustness of model predictions was analyzed in the following manner. We simulated villous area and antibody level during different treatments taking the value of model parameters from the confidential intervals identified using method described in section “Simulations on the range of parameter’s confidential intervals” (Additional file 1). Results are presented in Additional file 1: Figure S15-24. From the analysis we can conclude that model predictions of influence of TG-2 inhibitor, permeability inhibitor, DQ-2 blocking peptides on villous are and antibody level and IFN-γ, IL-15 antibodies on antibody level are quite robust to variation in model parameters. On the other hand, robustness of the model predictions for IFN-γ (Additional file 1: Figure S22) and IL-15 (Additional file 1: Figure S23) antibodies influence on villous area is not satisfactory. This can be explained in terms of lack of sufficient amount of available experimental data required for reliable identification of parameter values responsible for the processes associated with contribution of IFN-γ and IL-15 to immune response in CD. This pushes us to state that model of celiac disease developed in our manuscript can be applied to identify gaps in experimental and clinical data and to initiate more quantitative and rational exploration of the processes involved in CD pathogenesis. Taking these gaps in experimental/clinical data in mind model can advise on optimal design of clinical study or experiment [58,59].

## Conclusions

Overall, the use of antibodies against IFN-γ, and IL-15, analogues of gluten peptides repressing IEC activation, and TG-2 inhibitor would not be efficient solutions to treat CD. The model indicates that the most efficient therapeutic agent is a product that acts on species involved in both the innate and the adaptive immune response, i.e. analogues of gluten peptides that arrest APC activation. This model could be applied in the pharmaceutical R&D arena for the design of drugs against autoimmune small intestine disorders and on the design of their corresponding clinical trials.

## Abbreviations

CD: Celiac disease; TG-2: Transglutaminase-2; GFD: Gluten free diet; QSP: Quantitative systems pharmacology; IEC: Intestinal epithelial cells; IEL: Intraepithelial lymphocytes, aka. natural killers; IL-15: Interleukin-15; IL-21: Interleukin-21; IFN-γ: Interferon γ; IF-21: Single variable that represents both IFN-γ and IL-21; APC: Antigen-presenting cell.

## Competing interests

The authors declare that they have no competing interests.

## Authors’ contributions

OOD developed celiac disease immune response model, verified it and ran model simulations; SVS participated in model development and verification; VVS participated in model verification and ran model simulations; LC, CP, MVF, NB and OVD participated in study design and coordination and helped to draft the manuscript. All authors read and approved the final manuscript.

## Supplementary Material

Additional file 1 **Detailed description of the model system of differential equations, rate laws and model verification.** List of model parameters and variables. Click here for file
