# Supplementary material for: Modeling of celiac disease immune response and the therapeutic effect of potential drugs
Source: BMC Syst Biol. 2013 Jul 5;7:56. doi: 10.1186/1752-0509-7-56 (PMC3706242; doi:10.1186/1752-0509-7-56)
Supplement: Additional file 1 — Detailed description of the model system of differential equations, rate laws and model verification. List of model parameters and variables. [file 1752-0509-7-56-S1.docx]

**Supplementary materials**

**ODE system**

Ordinary differential equations describing dynamics and regulations of set of the reactions shown in Fig. S1 can be written down in the following manner: The abbreviations used are described in Table 1.

$$\frac{dG_{gut}}{dt}=(V_{1}-V_{2}-V_{13})/V_{lum}$$

$$\frac{dIEC}{dt}=(V_{3}-V_{4}-V_{5}+V_{27})/V_{lam}$$

$$\frac{dIEC_{a}}{dt}=(V_{5}-V_{6}-V_{27})/V_{lam}$$

$$\frac{dIL15}{dt}=(V_{7}-V_{8})/V_{lam}$$

$$\frac{dIEL}{dt}=(V_{9}-V_{10}-V_{11})/V_{lam}$$

$$\frac{dIEL_{a}}{dt}=(V_{11}-V_{12})/V_{lam}$$

$$\frac{dG_{muc}}{dt}=(V_{13}-V_{14}-V_{29}-V_{33})/V_{lam}$$

$$\frac{dG_{d}}{dt}=(V_{33}-V_{15}-V_{30})/V_{lam}$$

$$\frac{dAPC}{dt}=(V_{16}-V_{17}-V_{18})/V_{lam}$$

$$\frac{dAPC_{a}}{dt}=(V_{18}-V_{19})/V_{lam}$$

$$\frac{dT}{dt}=(V_{20}-V_{21}-V_{22})/V_{lam}$$

$$\frac{dT_{i}}{dt}=(V_{22}-V_{34})/V_{lam}$$

$$\frac{dIF21}{dt}=(V_{23}-V_{24})/V_{lam}$$

$$\frac{dAb}{dt}=(V_{25}-V_{26}-V_{29}-V_{30})/V_{lam}$$

$$\frac{dT_{a}}{dt}=(V_{34}-V_{28})/V_{lam}$$

$$\frac{dZ\ln}{dt}=(V_{31}-V_{32})/V_{lum}$$

The abbreviations used are described in Table 1. The model describes processes that take place in two intestinal compartments (lumen and lamina propria) and incorporates 16 variables and 34 processes, which correspond to 34 reaction rates.

**Reaction rates**

This section describes the reaction rates of each of the processes shown in Fig. S1.

The influx rate of gluten proteins into the lumen ($V_{1}$), the maturation rate of IELs ($V_{9}$) and T-cells ($V_{20}$) are given by zero order rate laws:

$$V_{20}=V_{lam}\cdot(k_{m}^{t})$$

$$V_{9}=V_{lam}\cdot(k_{m}^{iel})$$

$$V_{1}=V_{lum}\cdot(k_{\inf lux})$$

Reaction rates of this kind correspond to a constant influx of peptides and recruitment of cells into the corresponding compartment.

The degradation rates of gluten peptides in the lumen ($V_{2}$); the breakdown rates of: IL-15 ($V_{8}$), native ($V_{14}$) and deamidated ($V_{15}$) gluten peptides, IF-21 ($V_{24}$), antibodies ($V_{26}$) in the lamina and zonulin in the lumen ($V_{32}$); the synthesis rate of activated IECs ($V_{31}$) and the death rate of non-activated ($V_{17}$) and activated ($V_{19}$) APCs, non-activated ($V_{21}$) and activated ($V_{28}$) T-cells; the rate of inactivation of IECs ($V_{27}$) and one of the stages of T-cell activation ($V_{34}$) are given by first order reaction rates:

$$V_{34}=V_{lam}\cdot(k_{a}^{i}\cdot T_{i})$$

$$V_{31}=V_{lam}\cdot(k_{s}^{z\ln}\cdot IEC_{a})$$

$$V_{32}=V_{lum}\cdot(k_{d}^{z\ln}\cdot Z\ln)$$

$$V_{28}=V_{lam}\cdot(k_{d}^{t}\cdot T_{a})$$

$$V_{26}=V_{lam}\cdot(k_{d}^{ab}\cdot Ab)$$

$$V_{27}=V_{lam}\cdot(k_{ia}\cdot IEC_{a})$$

$$V_{24}=V_{lam}\cdot(k_{d}^{if21}\cdot IF21)$$

$$V_{19}=V_{lam}\cdot(k_{d}^{apc}\cdot APC_{a})$$

$$V_{21}=V_{lam}\cdot(k_{d}^{t}\cdot T)$$

$$V_{17}=V_{lam}\cdot(k_{d}^{apc}\cdot APC)$$

$$V_{15}=V_{lam}\cdot(k_{d}^{deam}\cdot G_{d})$$

$$V_{14}=V_{lam}\cdot(k_{muc}^{d}\cdot G_{muc})$$

$$V_{8}=V_{lam}\cdot(k_{d}^{il15}\cdot IL15)$$

$$V_{2}=V_{lum}\cdot(k_{d}^{gut}\cdot G_{gut})$$

Equations of this kind describe processes that take place at rates proportional to the concentration of peptides, proteins, or cells.

The maturation rate of IEC ($V_{3}$) is given by the following equation:

$$V_{3}=V_{lam}\cdot(k_{m}^{iec}\cdot\frac{1}{1+\frac{Ab}{K_{d\_inh}^{iec}}})$$

In the absence of antibodies, (i.e. when Ab = 0), the maturation rate of IEC remains constant. However, with the increase of antibody levels, the production rate of IEC declines.

The death rates of activated ($V_{6}$) and non-activated IECs ($V_{4}$) are given by two equations, the right hand side of which is proportional to the level of activated and non-activated IECs:

$$V_{6}=V_{lam}\cdot(k_{d}^{iec}\cdot IEC_{a}\cdot(1+\frac{Ab}{K_{d}^{ab}}+\frac{IEL_{a}}{K_{d}^{iel}}+\frac{IF21}{K_{d}^{if21}})$$

$$V_{4}=V_{lam}\cdot k_{d}^{iec}\cdot IEC\cdot(1+\frac{Ab}{K_{d}^{ab}}+\frac{IEL_{a}}{K_{d}^{iel}}+\frac{IF21}{K_{d}^{if21}})$$

It can be seen that the promotion of IEC death is a function of the level of antibodies, activated IELs, IFN-γ, IL-21 and zonulin. The contribution of an increase in any of these levels to the total death rate is given by the fractional rational function in the right hand side of the equation. If one of the above-mentioned agents is absent, then the factor that represents its contribution takes the value of zero. In general, antibodies, activated IELs, IFN-γ and IL-21 work additively to induce the apoptosis of IECs.

The IEC activation rate by gluten peptides is linearly dependent on the concentration of non-activated IECs:

$$V_{5}=V_{lam}\cdot(k_{a}^{iec}\cdot IEC\cdot(\frac{G_{gut}}{K_{d}^{iec}})^{p}\cdot\frac{1-(\frac{G_{gut}}{K_{d}^{iec}})^{n-p+1}}{1-(\frac{G_{gut}}{K_{d}^{iec}})^{n+1}})$$

This expression includes an S-shaped dependence of the IEC activation rate on gluten peptides in the lumen, which is in agreement with the presence of a threshold level of IEC activation. In fact, IECs can never become activated until a certain fraction of CXCR3 receptors in IECа cells have been occupied with gluten proteins. The relation between the parameters *n* and *p* in the equation describes the ratio of the total CXCR3 receptors in IECа cells to the receptor number required for IEC activation.

The IL-15 synthesis rate ($V_{7}$) by activated IECs and APCs and the IF-21 synthesis rate by IELs and Т-cells ($V_{23}$) are given by the following equations:

$$V_{23}=V_{lam}\cdot(k_{s\_1}^{if21}\cdot IEL_{a}+k_{s\_2}^{if21}\cdot T_{a})$$

$$V_{7}=V_{lam}\cdot(k_{s\_iec}^{il15}\cdot IEC_{a}+k_{s\_apc}^{il15}\cdot APC_{a})$$

The death rate of activated ($V_{12}$) and non-activated ($V_{10}$) IELs is proportional to their concentration:

$$V_{10}=V_{lam}\cdot(k_{d}^{iel}\cdot IEL\cdot\frac{1}{1+\frac{IL15}{K_{d\_inh}^{2}}})$$

$$V_{12}=V_{lam}\cdot(k_{d}^{iel}\cdot IEL_{a}\cdot\frac{1}{1+\frac{IL15}{K_{d\_inh}^{2}}})$$

Both processes are inhibited by IL-15, which is taken into account via the corresponding fractional rational function. The increase of IL-15 levels leads to the decrease of the IEC production rate.

The activation rate of IELs by IL-15 (IL15) is given by the following equation:

$$V_{11}=V_{lam}\cdot(k_{a}^{iel}\cdot IL15\cdot\frac{IEL}{IL15+K_{d}^{1}})$$

The rate at which gluten peptides arrive in the lamina propria from the lumen is described by a reversible process, i.e. gluten peptides can enter the lamina from the lumen and vice versa, depending on a concentration gradient, as described below:

$$V_{13}=V_{lum}\cdot(k_{t}^{g}\cdot((1-(\frac{IEC_{tot}}{0.0085})^{3})+Permeability)\cdot(G_{gut}-G_{muc}))$$

where

$$Permeability=1-\frac{1+\frac{k^{z\ln}\cdot Z\ln}{K_{d}^{z\ln}}}{1+\frac{Z\ln}{K_{d}^{z\ln}}}$$

Here $IEC_{tot}$ is the total concentration of activated and non-activated IECs. In a healthy individual this concentration is equal to 0.0085 pM (see “Parameters identification against steady-state *in vivo* data” below). The equation illustrates how IEC numbers and Zonulin influence on the invasion of gluten peptide into the lamina propria. $k^{z\ln}$ is less than 1, so increase in Zonulin level will lead to Permeability decrease and velocity increase. Increase in total level of IECs results in velocity decrease.

The recruitment rate of APCs is stimulated by IL-15 since it induces differentiation of dendritic cells and monocytes into APCs:

$$V_{16}=V_{lam}\cdot(k_{in}^{apc}\cdot(1+\frac{IL15}{K_{d\_stim}^{il15}}))$$

If the IL-15 level is zero, then the recruitment rate of APC is constant. As the IL-15 level increases, the recruitment rate of APCs will rise.

The activation rate of APCs is proportional to their concentration:

$$V_{18}=V_{lam}\cdot(k_{a}^{apc}\cdot APC\cdot(\frac{TG\_G}{EC_{50}^{tg\_g}+TG\_G}+\frac{G_{d}}{EC_{50}^{deam}+G_{d}}+\frac{G_{muc}}{EC_{50}^{muc}+G_{muc}}))$$

and depends on the antigen load (deamidated and non-deamidated gluten peptide as well as gluten peptide complexed to TG-2) and its EC50 for APC activation. The contributions of various antigens to APC activation were assumed additive.

The activation rate of Т-cells is directly proportional to their level and to the concentration of activated APCs:

$$V_{22}=V_{lam}\cdot(k_{a}^{t}\cdot T\cdot APC_{a}\cdot(1+\frac{IF21}{K_{d\_stim}^{act}}))$$

IF-21 (IFN-γ + IL-21) stimulates Т-cell activation, but only if the concentration of activated APCs is higher than zero.

The antibody synthesis rate is dependent on antigen concentration and its ЕС50 for APC activation, and is stimulated by activated-cells:

$$V_{25}=V_{lam}\cdot(k_{s}^{ab}\cdot(1+\frac{T_{a}\cdot k_{ab}^{ta}}{K_{d\_ab}^{ta}+T_{a}})\cdot(\frac{TG\_G}{EC_{50}^{tg\_g}+TG\_G}+\frac{G_{d}}{EC_{50}^{deam}+G_{d}}+\frac{G_{muc}}{EC_{50}^{muc}+G_{muc}}))$$

The rate of antibody binding with antigens (native ($V_{29}$) and deamidated ($V_{30}$) peptides) followed by the degradation of their complex is given by the following equations:

$$V_{29}=V_{lam}\cdot(\frac{k_{d}^{ag\_ab}\cdot G_{muc}\cdot Ab}{K_{d}^{ab\_2}})$$

$$V_{30}=V_{lam}\cdot(\frac{k_{d}^{ag\_ab}\cdot k_{rat}\cdot G_{deam}\cdot Ab}{K_{d}^{ab\_2}})$$

These reaction rates were derived from 2 reactions describing antigen-antibody complex formation and its elimination. Parameter $k_{d}^{ag\_ab}$ describes the half-life of antigen-antibody complex, parameter $K_{d}^{ab\_2}$ describes affinity of antibody to antigen, parameter $k_{rat}$ describes the fact that deamidated peptides have greater affinity to antibodies that native peptides [1].

The deamidation rate of gluten peptides, i.e. the reaction rate of TG-2, is given by the Michaelis-Menten equation [2, 3]:

$$V_{33}=V_{lam}\cdot(\frac{k_{d}^{cat}\cdot e_{0}\cdot G_{muc}\cdot\frac{1}{1+\frac{Ab}{K_{d}^{e\_ab}}}}{K_{m}^{deam}+G_{muc}})$$

where $e_{0}$ is the total concentration of TG-2. This reaction takes into consideration that antibodies decrease the enzyme activity, since TG-2 is an antigen.

The TG-2 complex and gluten peptide concentration (TG_G) was described with a function defining a concentration of an enzyme-substrate complex that is derived in the context of a Michaelis-Menten equation derivation [2, 3]:

$$TG\_G=\frac{\frac{e_{0}\cdot G_{muc}}{1+\frac{Ab}{K_{d\_e}^{ab}}}}{K_{m\_deam}+G_{muc}}$$

This function describes the fact that antibodies inactivate TG-2 when they bind to it.

To describe the dependence of the small intestinal villous area on the total concentration of IECs, the following empirical function was introduced:

$$VA=((k_{va})^{(1-\frac{IEC_{tot}}{0.0085})}\cdot(\frac{IEC_{tot}}{0.0085})^{4})\cdot100\%$$

Here, $IEC_{tot}$ is the total concentration of activated and non-activated IEC, 0.0085 pM is the concentration of IECs in a healthy human (see “Parameters identification against steady-state *in vivo* data” below). This empirical function, which describes the percentage of the small intestinal villous area and is expressed through a cumulative concentration of activated and non-activated IECs, was derived assuming the following conditions: i) the value of this function should range from 0% (when there is not any IECs) to 100% (when total IECs concentration is 0.0085 pM), and ii) it should describe experimental data (see “Identification of Model Parameters” below).

**Estimation of compartmental volumes**

This model considers two separate compartments located in the small intestine: the intestinal lumen and the lamina propria. The lumen is the cavity where digested food passes through and from where nutrients are absorbed. The lamina propria is a thin layer of loose connective tissue that lies beneath the intestinal epithelium, and together with the epithelium constitutes the mucosa. The volumes of these compartments were estimated from the following experimental data:

(1) The length of the small intestine ranges between 3 and 7 meters, and the diameter of the luminal passage is ca. 2.5-3 cm [4, 5, 6].

(2) The length of intestinal villi is ca. 0.5-1.5 mm [7].

(3) Using micrography methods, Guix et al [8] determined that the thickness of the lamina compartment is about 1.5-3 mm, with the assumption that villi are tightly packed and form a single and compact lamina layer.

The volume of the small intestine consists of the lumen volume ($V_{lum}$) and the lamina volume ($V_{lam}$). It was assumed that the intestine has approximately the shape of a cylinder of radius *R* (sum of lumen radius and lamina thickness) and length *L* (see Fig. S2). Similarly, the lumen could be described as a cylinder of radius *r* and length *L*. Therefore the volume of lamina and lumen could be calculated as follows:

$$V_{lum}=\pi\cdot r^{2}\cdot L$$

$$V_{lam}=\pi\cdot(R^{2}-r^{2})\cdot L$$

Assuming that the small intestinal length and radius are *L* = 7 m and *R* = 18 mm (experimental evidence (1) and (3)), and the lumen diameter is *r* = 15 mm (experimental evidence (1)), the resulting compartmental volumes were $V_{lum}$= 4.95 liters and $V_{lam}$= 2.18 liters.

**Parameter identification against in vitro and ex vivo data**

A set of parameters was verified against *in vitro* and *ex vivo* data, involving the following model components:

1. IFN-γ synthesis by T-cells [9] (Fig. S3). This allowed to identify 1 parameter ($k_{k\_2}^{if21}$ for IFN-γ).
2. IL-21 synthesis by T-cells [10] (Fig. S4). This allowed to identify 1 parameter ($k_{k\_2}^{if21}$ for IL-21).
3. IL-15 degradation [11] (Fig. S5). This allowed to identify 1 parameter ($k_{d}^{il15}$).
4. IEL death [12] (Fig. S6). This allowed to identify 1 parameter ($k_{d}^{iel}$).
5. IEL death [12] (Fig. S7). This allowed to identify 1 parameter ($K_{d\_inh}^{iel}$).
6. IFN-γ synthesis by IELs [13] (Fig. S8). This allowed to identify 1 parameter ($k_{s\_1}^{if21}$).
7. IEC death [14] (Fig. S9). This allowed to identify 1 parameter ($k_{d}^{iec}$).
8. Peptide transport from the lumen to the lamina through the small intestinal epithelium in patients sustained on gluten-free and gluten containing diet [15] (Fig. S10). This allowed to identify 1 parameter ($k_{t}^{g}$).
9. T-cell death [16] (Fig. S11). This allowed to identify 1 parameter ($k_{d}^{t}$).
10. Inhibition of TG-2 by anti-TG antibodies [17, 18] (Fig.S12). This allowed to identify 1 parameter ($K_{d\_ab}^{e}$).
11. Zonulin influence on permeability [19,20,21] (Fig. S13). This allowed to identify 2 parameters ($K_{d}^{z\ln}$,$k^{z\ln}$)

**Parameter identification against steady-state in vivo data**

Some of the remaining parameters were determined using steady-state simulations in the following way:

- Those parameters validated against *in vitro* data and taken from literature, were fixed and were not changed.
- Other parameters were changed so that the concentrations of certain substances at steady-state coincided with specific values taken from literature.
- The model was verified against three different steady states: i) a healthy patient (simulated as a CD patient that has never taken in gluten-containing food products); ii) a patient on GFD (a CD person that consumes food products with low gluten content); iii) a patient on a gluten-containing diet (a CD person that consumes food products with normal gluten content).

Steady-state concentrations of cells (APCs, IELs, IECs, T cells) and peptides/proteins (IL-15, Zonulin, Antibodies) were used to identify model parameters. The APC levels and peptides/proteins concentrations at steady-state were taken from the literature [13, 22, 23, 24]. Other cellular steady-state concentrations were assessed as follows:

(1) IEL and T-cell numbers per surface area of the small intestine excluding villous area were taken from the literature [25].

(2) Using data on human anatomy (the length and radius of the small intestine: see section “Estimation of volumes of compartments”) the total area of the intestine was calculated.

(3) Then, with the lamina volume known, (see section “Estimation of volumes of compartments”) the IEL and T-cell numbers per lamina unit volume were obtained.

(4) The IEC concentration in the lamina was derived from literature measurements of IEL levels per 100 IECs [26, 27]

(5) As the lamina volume was known (see section “Estimation of volumes of compartments”), the absolute levels of IEL, T-cell, and IEC could be derived for the three categories of patients: a healthy patient, a CD patient on GFD, and a CD patient on a gluten-containing diet.

Assuming that in a healthy patient there are no both innate and adaptive immune responses, concentrations of activated cells, cytokines, antibodies, and zonulin are zero, and knowing the concentrations of IECs, IELs, T cells, APCs (see Table 3), and the rate constants of their apoptosis, the maturation rates of these cells could be determined. Consequently, 4 parameters were determined (see Table 2).

In the case of a patient on GFD, the inflow of gluten peptides into the small intestinal lumen was set to 750 nM per hour, which corresponds to the average inflow of gluten peptides on a GFD. As for a patient on a gluten-containing diet and healthy subjects, the inflow was set to 750 µM per hour, which corresponds to the average inflow of gluten peptides on a gluten-containing diet. Since gluten peptides arrive in the small intestine with food, their concentrations oscillate throughout the day with respect to meal intake. To simplify the model, the inflow of gluten peptides was considered constant throughout the day and set to the average daily inflow rate assuming a patient takes three daily meals. Overall, the parameters were selected so that the concentrations were similar to the known steady-state concentrations in the corresponding diets (Table 4). Consequently, 20 parameters were determined (see Table 2).

The parameter that define the villous area as a function of the total concentration of IECs were identified against data on changes in the villous area in patients throughout the year after changing over from a gluten-containing diet to a gluten-free diet; as well as changes in the villous area in healthy patients [28] (Fig. S14). Consequently, one parameter was determined (see Table 2).

**Simulations on the range of parameter’s confidential intervals**

Simulations drawing on the range of parameter’s confidential intervals were done to analyze the robustness of model predictions. For each parameter 1000 values from confidential interval were obtained by Monte Carlo method. Using these random parameter’s values simulations were done for each parameter separately. So, about 30000 simulations for each end point and therapeutic agent were done (Fig.S15-S24).

**Figures**

**Figure S1**

Network of processes of innate and adaptive immune responses described in the model.


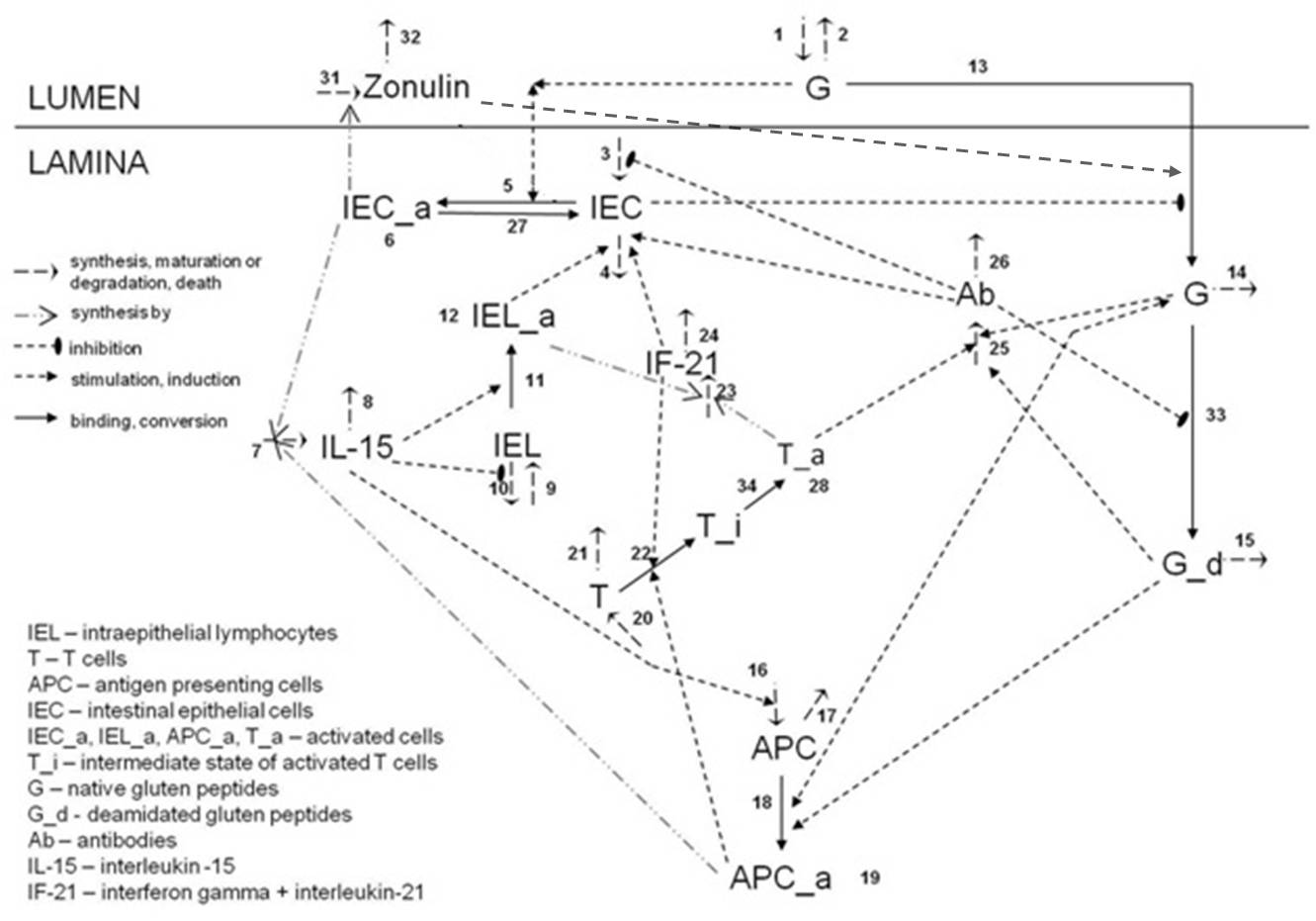


**Figure S2**

Small intestine, where L is length, R – radius of small intestine, r – radius of lumen of small intestine.


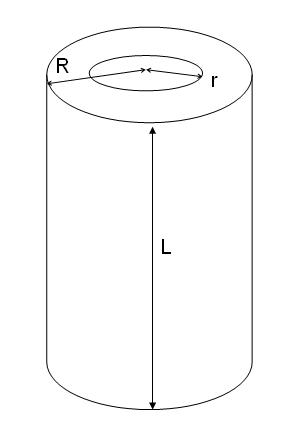


**Figure S3**

The IFN-γ synthesis by activated T-cells [9]. The experiment description: APCs and T-cells were incubated with gliadin. After a 48 h-incubation the IFN-γ load was measured. Overall, 1 parameter were determined.


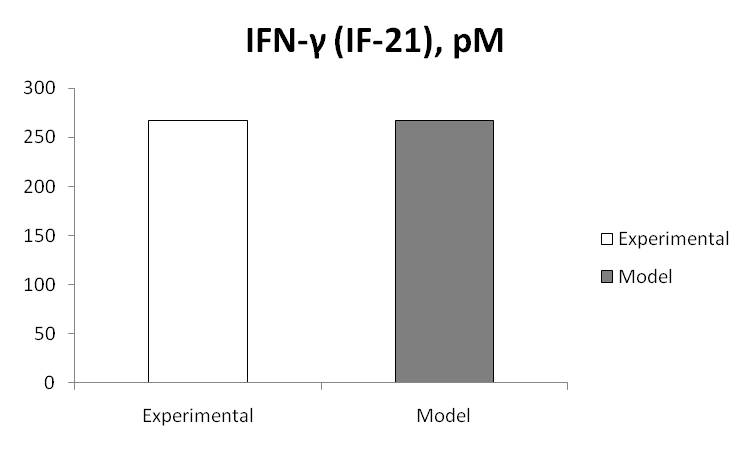


**Figure S4**

The IL-21 synthesis by activated T-cells [10]. The experiment description: activated T-cells were incubated and the IL-21 amount synthesized for 24 h was recorded. Overall, one parameter was determined.


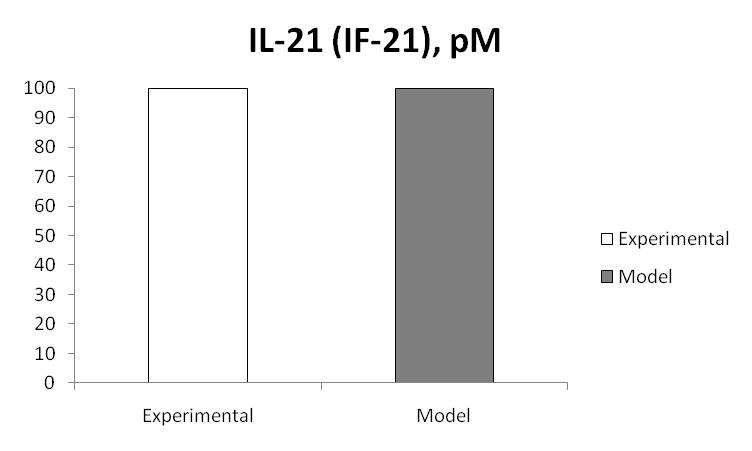


**Figure S5**

The IL-15 degradation [11]. The experiment description: IL-15 was incubated to look at its breakdown rate in a cellular solution. Overall, one parameter was determined.


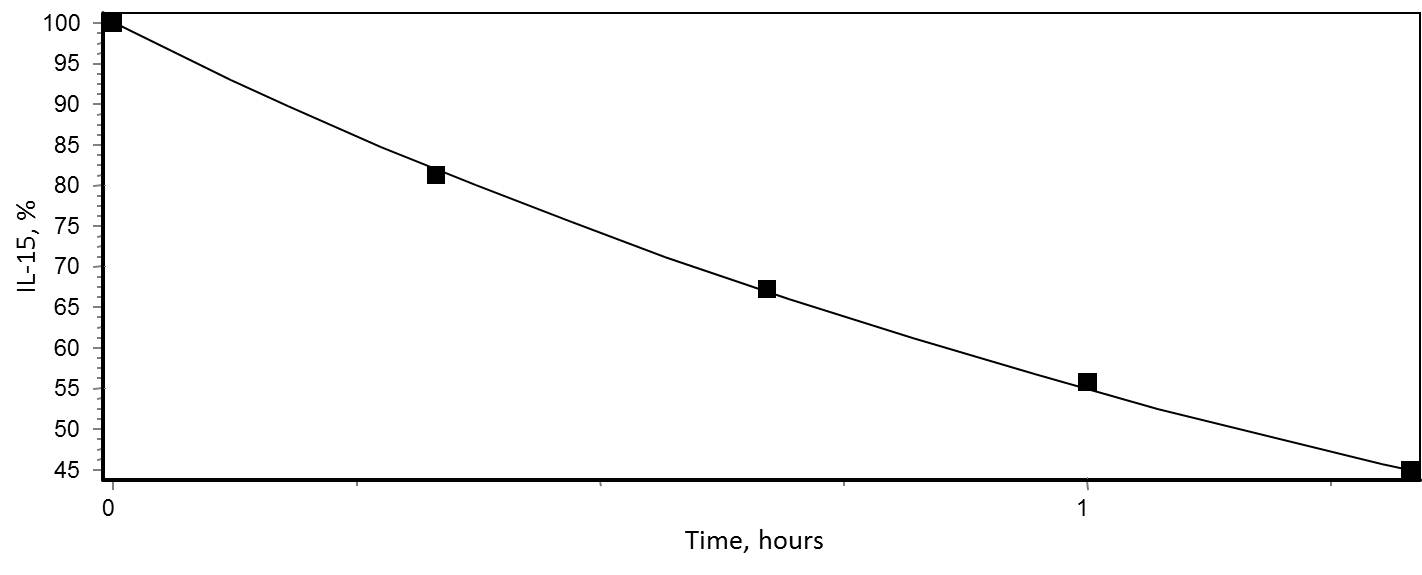


**Figure S6**

The IEL death [12]. The experiment description: IELs were incubated to look at their death. Overall, one parameter was determined.


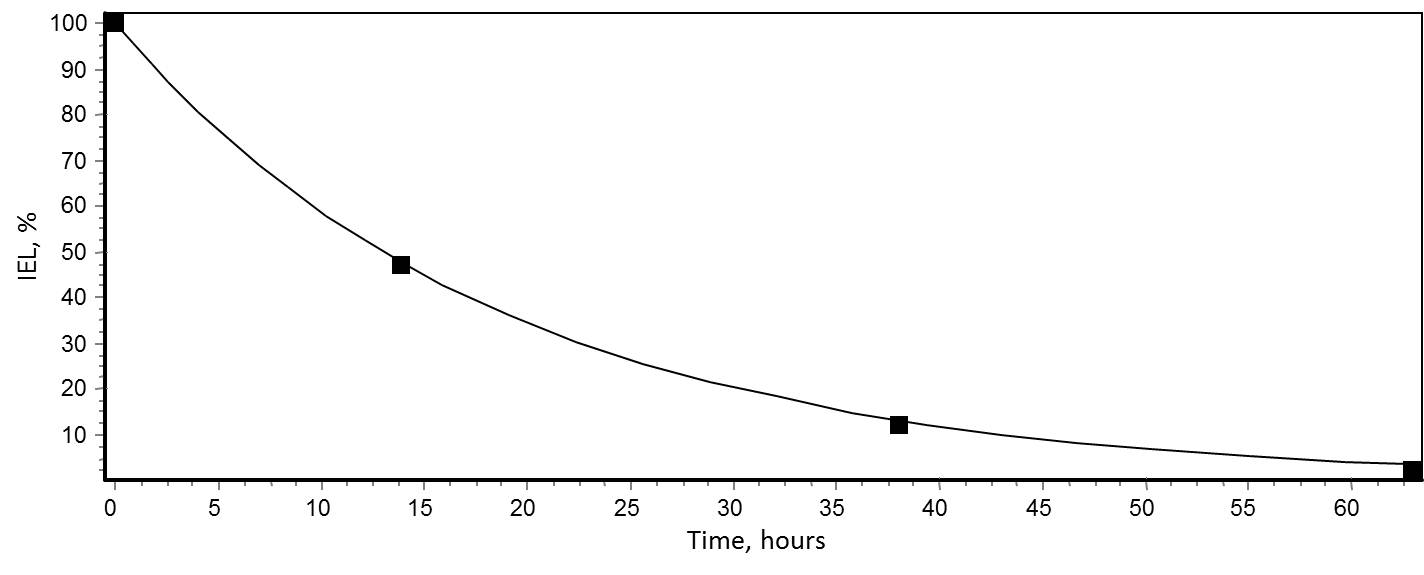


**Figure S7**

The IEL death [12]. The experiment description: IELs were incubated with IL-15 to look at their death. Overall, one parameter was determined.


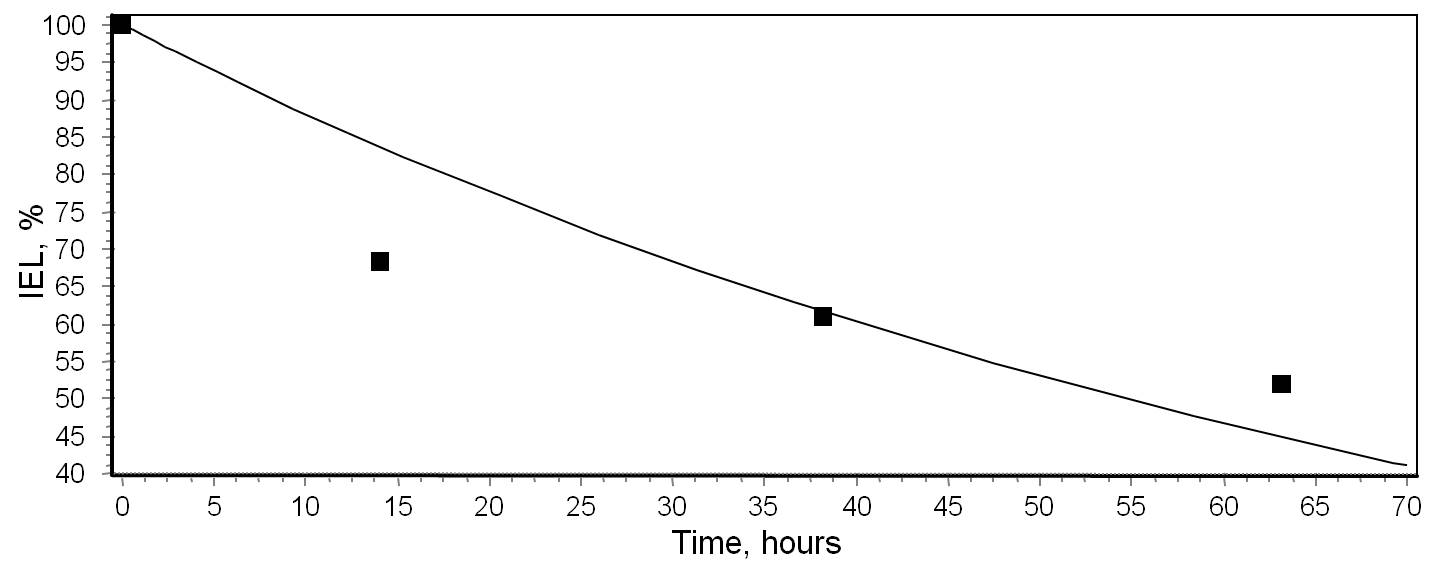


**Figure S8**

The IFN-γ synthesis by IELs [13]. The experiment description: IELs were incubated to monitor IFN-γ synthesis by activated IELs for 24 h. Overall, 5 parameters were determined.


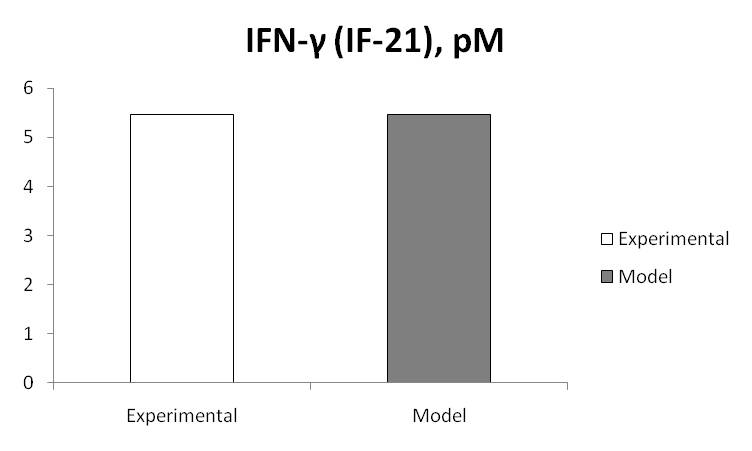


**Figure S9**

The IEC death [14]. The experiment description: IECs were incubated to monitor their death. Overall, one parameter was determined.


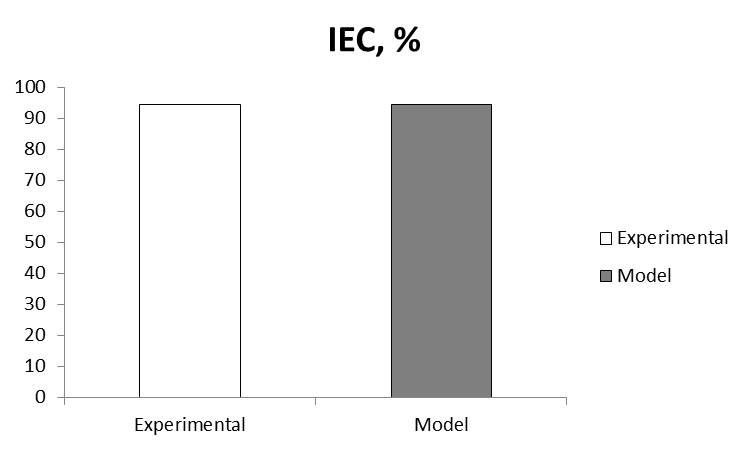


**Figure S10**

The transport of gluten peptides across the small intestinal epithelium [15]. The experiment description: biopsy samples from CD patients on gluten-containing (solid line) and GFD (dashed line) were evaluated for the amount of gluten peptides to move across the small intestinal epithelium for 3 h. For verification using these data, the known parameter was used: the degradation constant of gluten peptides. Overall, one parameter was determined.


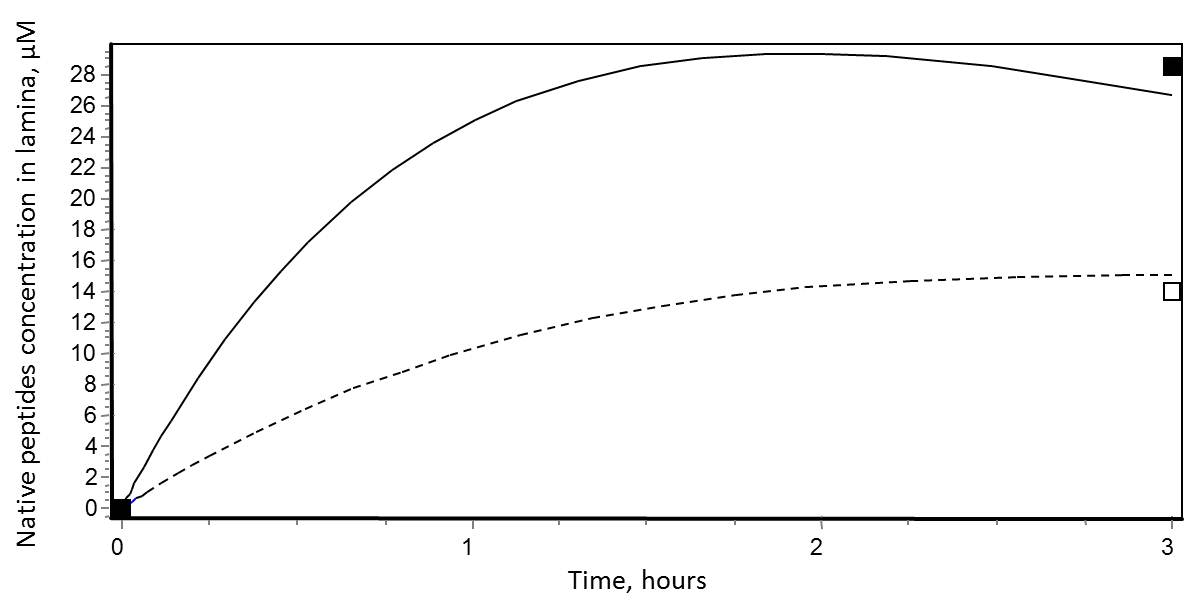


**Figure S11**

The Т-cell death [16]. The experiment description: T-cells were incubated to look at their death. Overall, one parameter was determined.


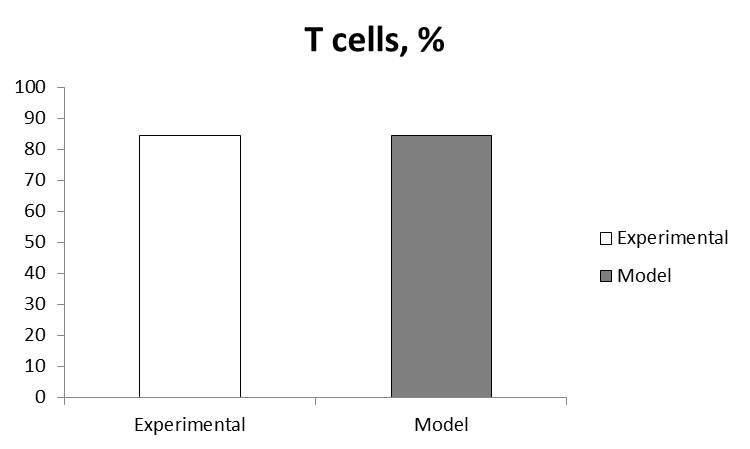


**Figure S12**

Inhibition of TG-2 by anti-TG antibodies [17, 18]. The experiment description: TG-2 were incubated with anti-TG antibodies to look at TG-2 activity. Overall, one parameter was determined.


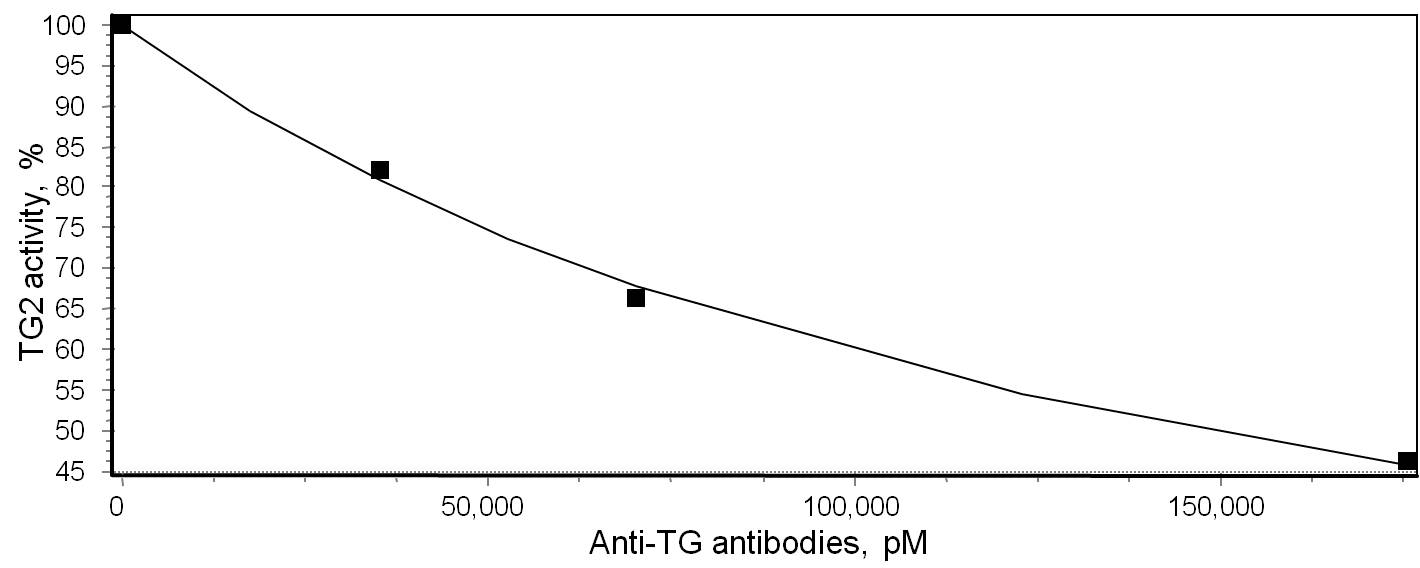


**Figure S13**

Zonulin influence on permeability [19, 20, 21]. The experiment description: Zonulin was incubated with intestinal epithelial cell lines to look at permeability. Overall, 2 parameter was determined.

**
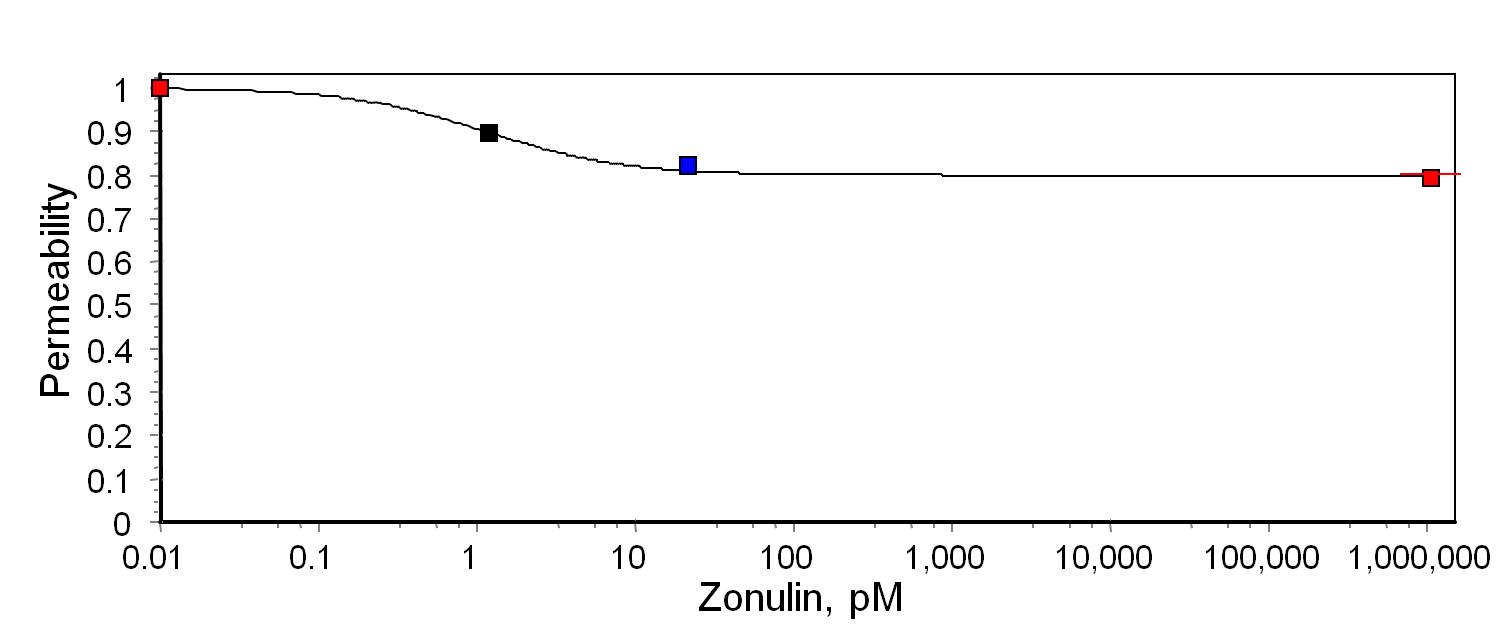
**

**Figure S14**

Verification of Villous Area function [28].


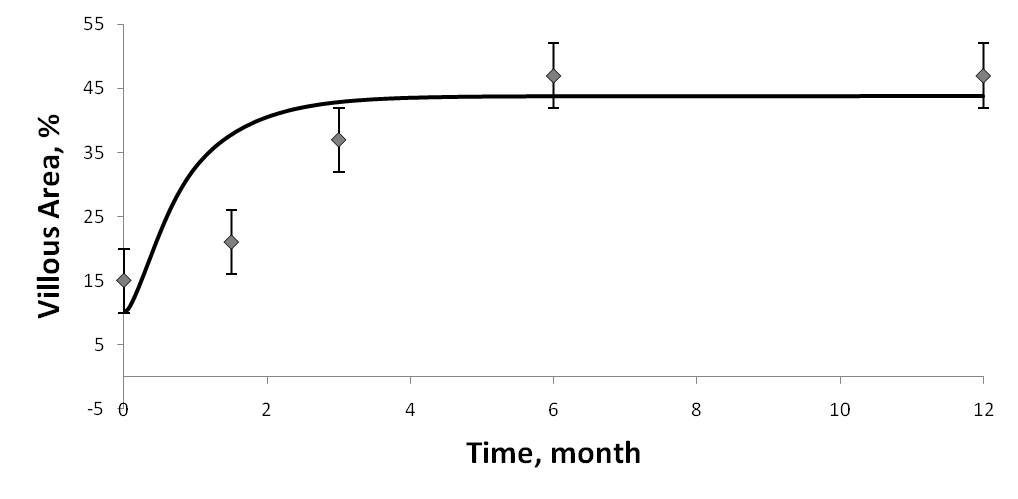


**Figure S15**

Simulation of DQ2-blocking peptide analogues action on Antibody level drawing on the range of parameter’s confidential intervals. Red curve – simulation with optimum value of parameters. Points – simulations with parameters values obtained by Monte Carlo method from confidential intervals.


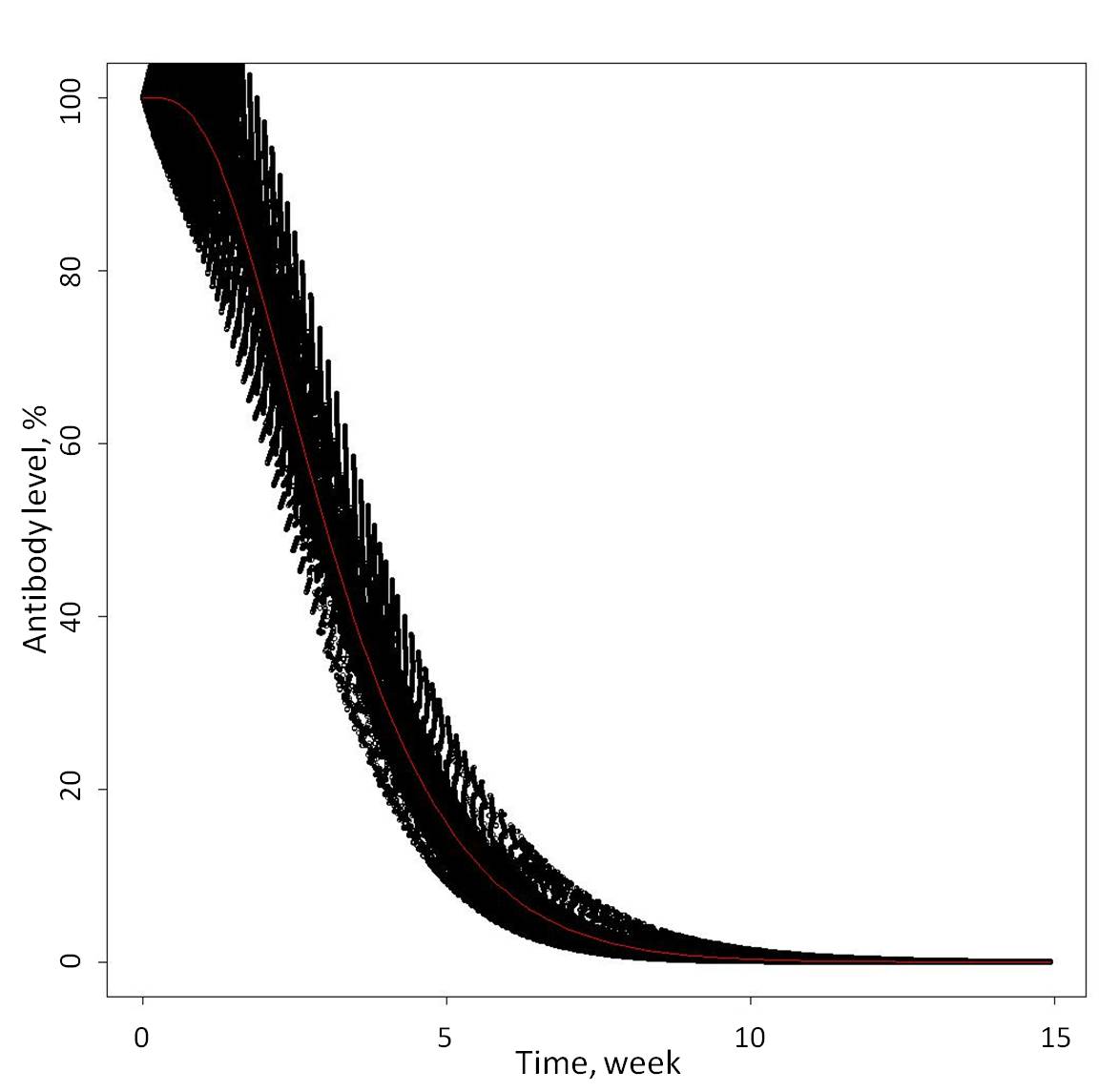


**Figure S16**

Simulation of Permeability inhibitor action on Antibody level drawing on the range of parameter’s confidential intervals. Red curve – simulation with optimum value of parameters. Points – simulations with parameters values obtained by Monte Carlo method from confidential intervals.


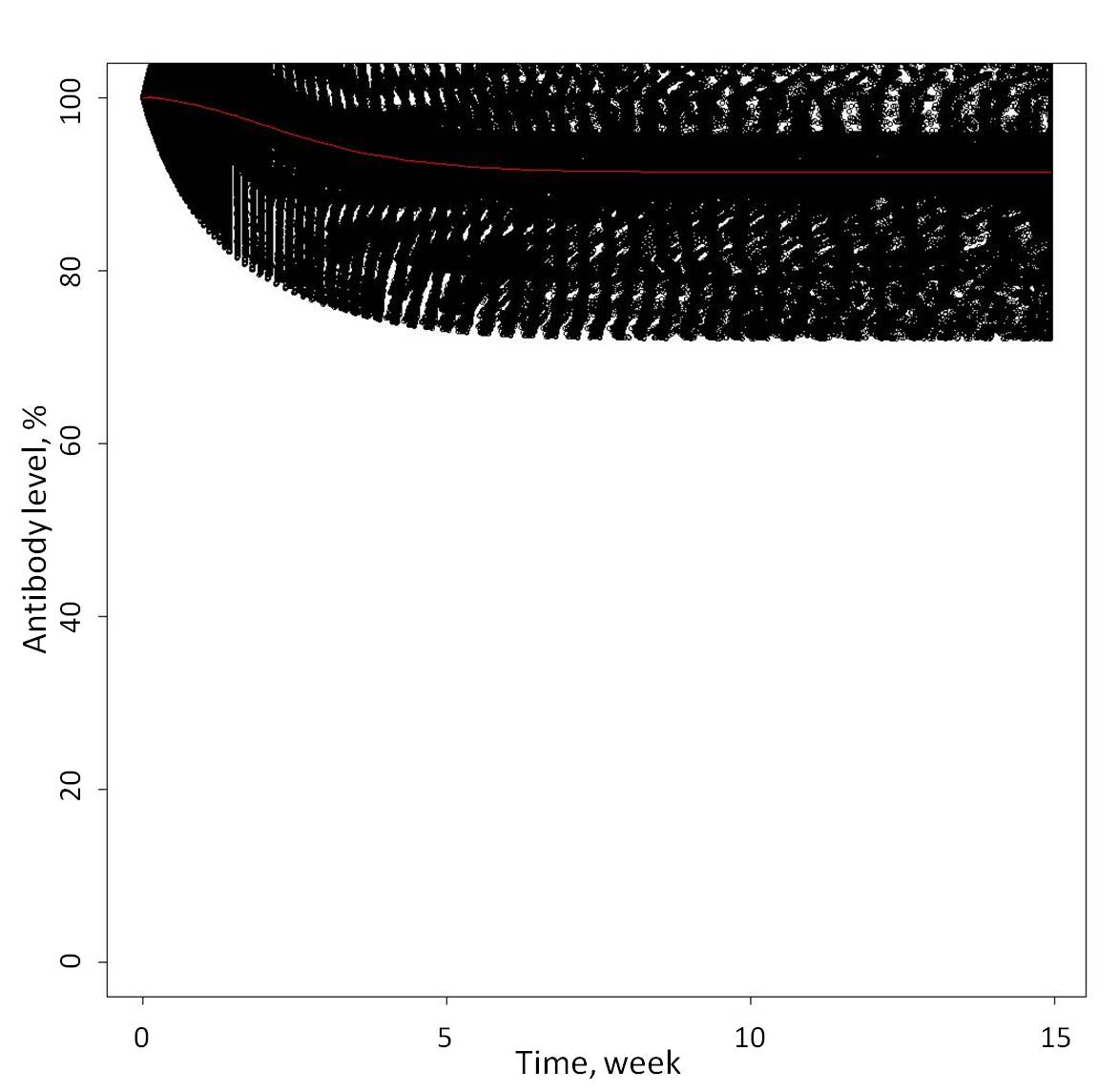


**Figure S17**

Simulation of IFN-γ antibodies action on Antibody level drawing on the range of parameter’s confidential intervals. Red curve – simulation with optimum value of parameters. Points – simulations with parameters values obtained by Monte Carlo method from confidential intervals.


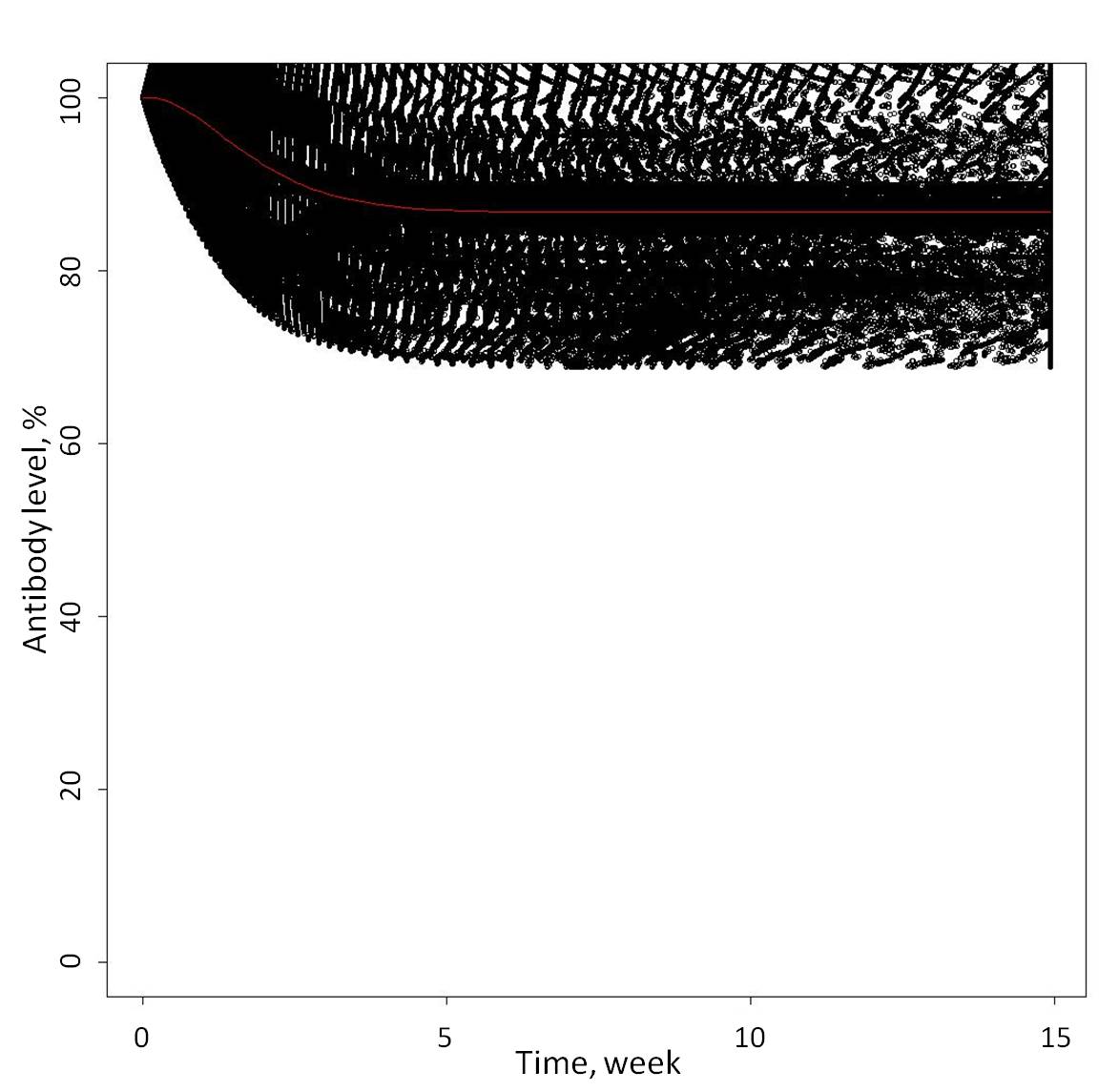


**Figure S18**

Simulation of IL-15 antibodies action on Antibody level drawing on the range of parameter’s confidential intervals. Red curve – simulation with optimum value of parameters. Points – simulations with parameters values obtained by Monte Carlo method from confidential intervals.


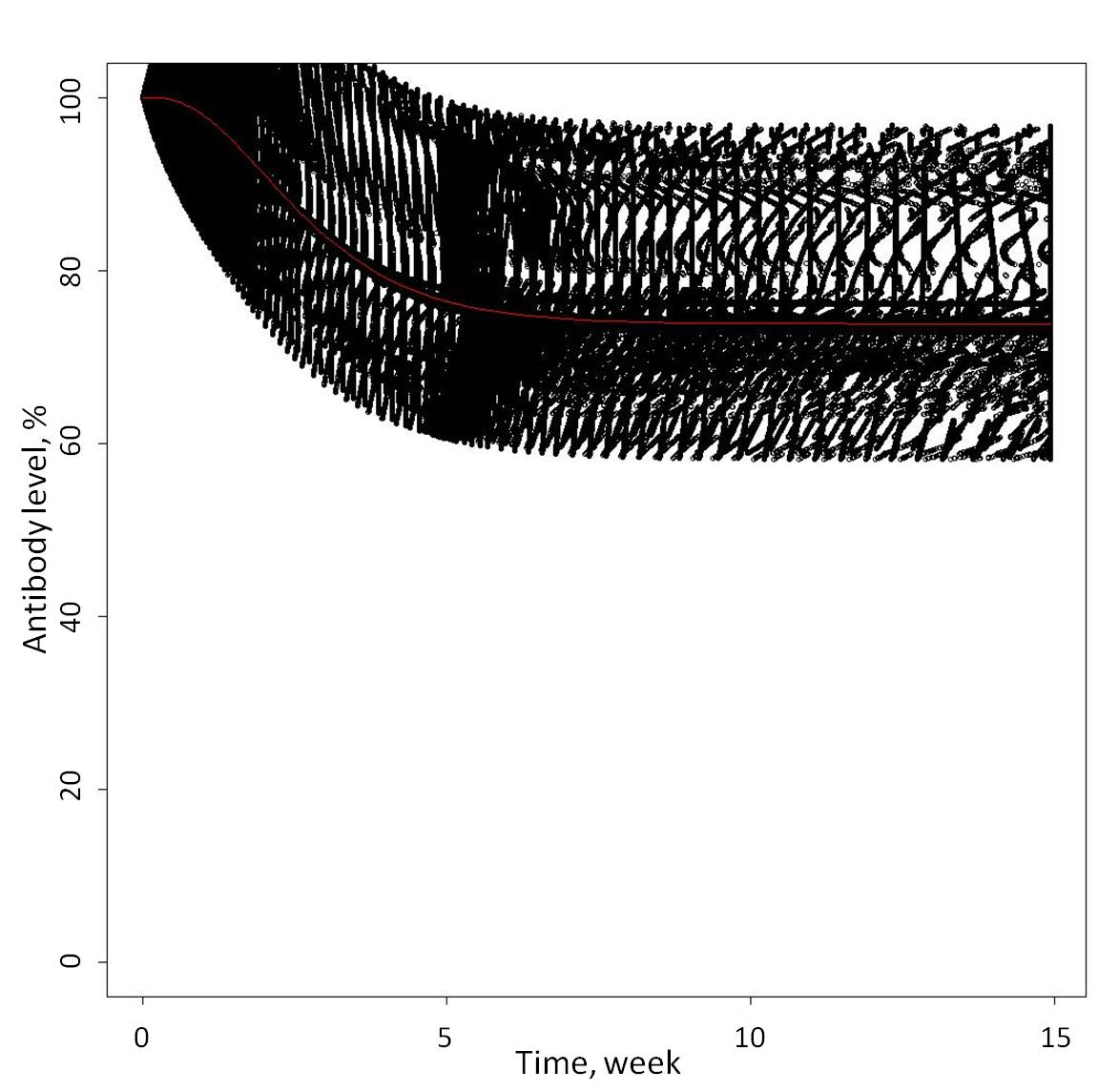


**Figure S19**

Simulation of TG-2 inhibitor action on Antibody level drawing on the range of parameter’s confidential intervals. Red curve – simulation with optimum value of parameters. Points – simulations with parameters values obtained by Monte Carlo method from confidential intervals.


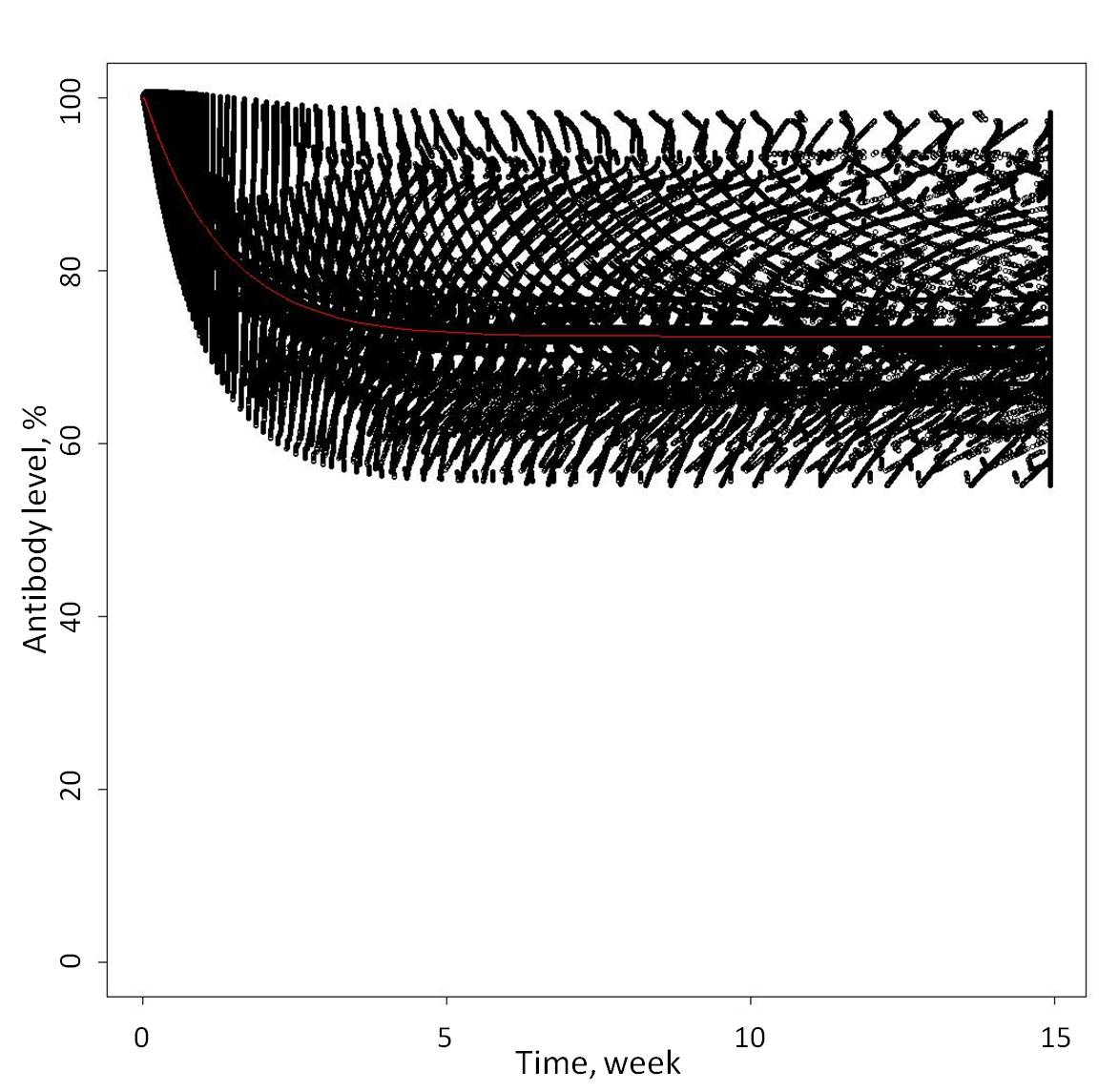


**Figure S20**

Simulation of DQ2-blocking peptide analogues action on Villous Area drawing on the range of parameter’s confidential intervals. Red curve – simulation with optimum value of parameters. Points – simulations with parameters values obtained by Monte Carlo method from confidential intervals.


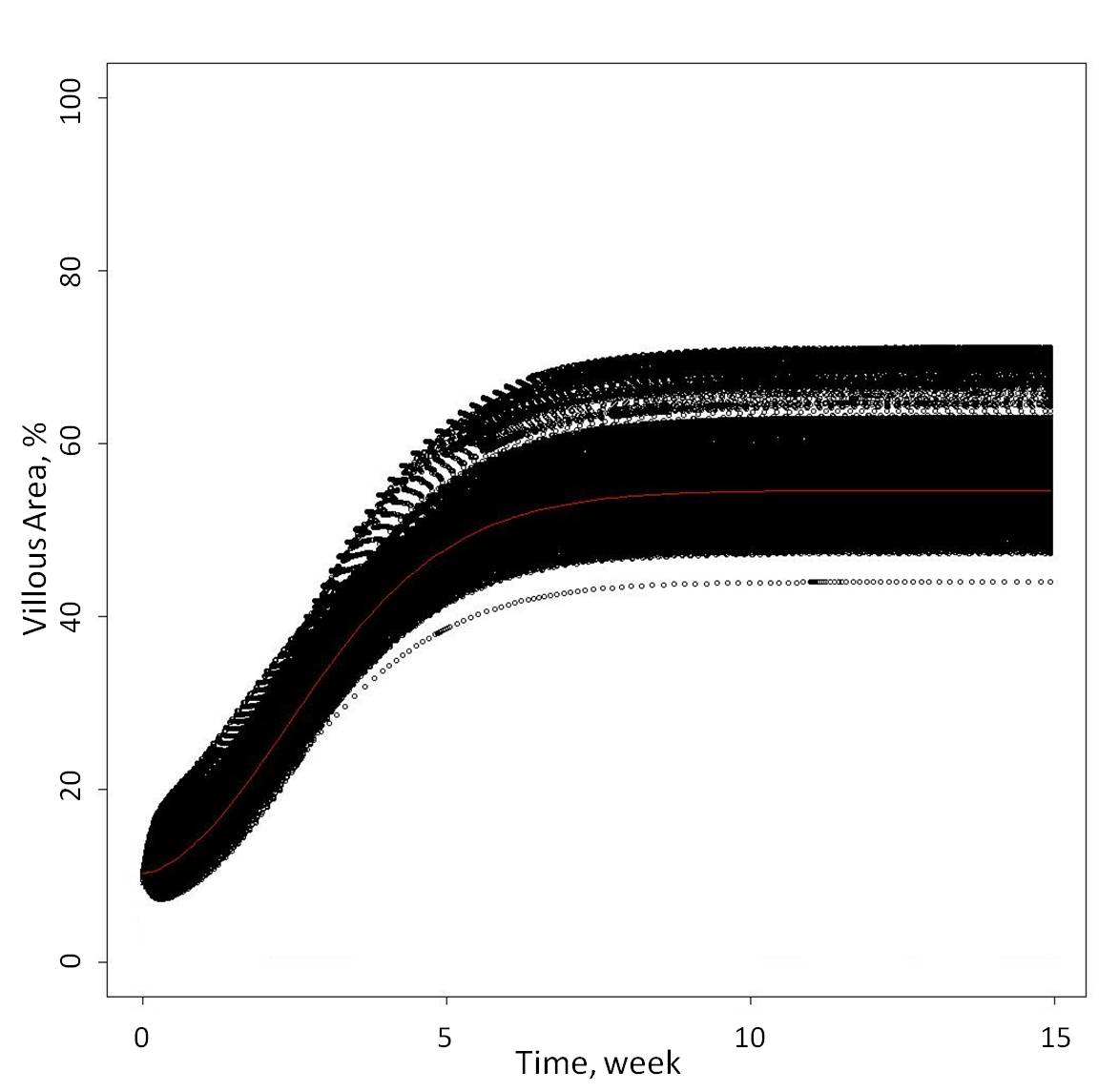


**Figure S21**

Simulation of Permeability inhibitor action on Villous Area drawing on the range of parameter’s confidential intervals. Red curve – simulation with optimum value of parameters. Points – simulations with parameters values obtained by Monte Carlo method from confidential intervals.
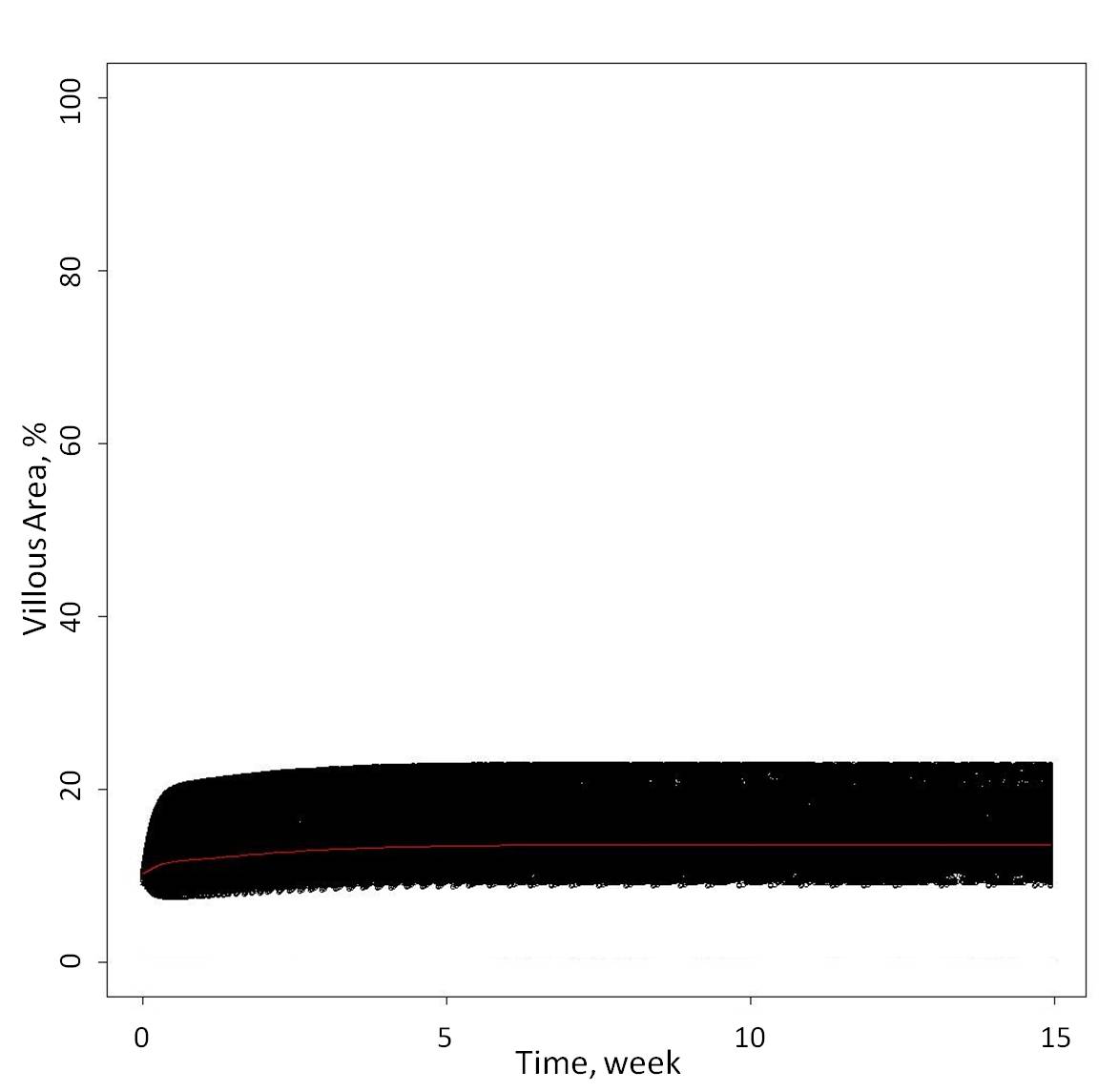


**Figure S22**

Simulation of IFN-γ antibodies action on Villous Area drawing on the range of parameter’s confidential intervals. Red curve – simulation with optimum value of parameters. Points – simulations with parameters values obtained by Monte Carlo method from confidential intervals.


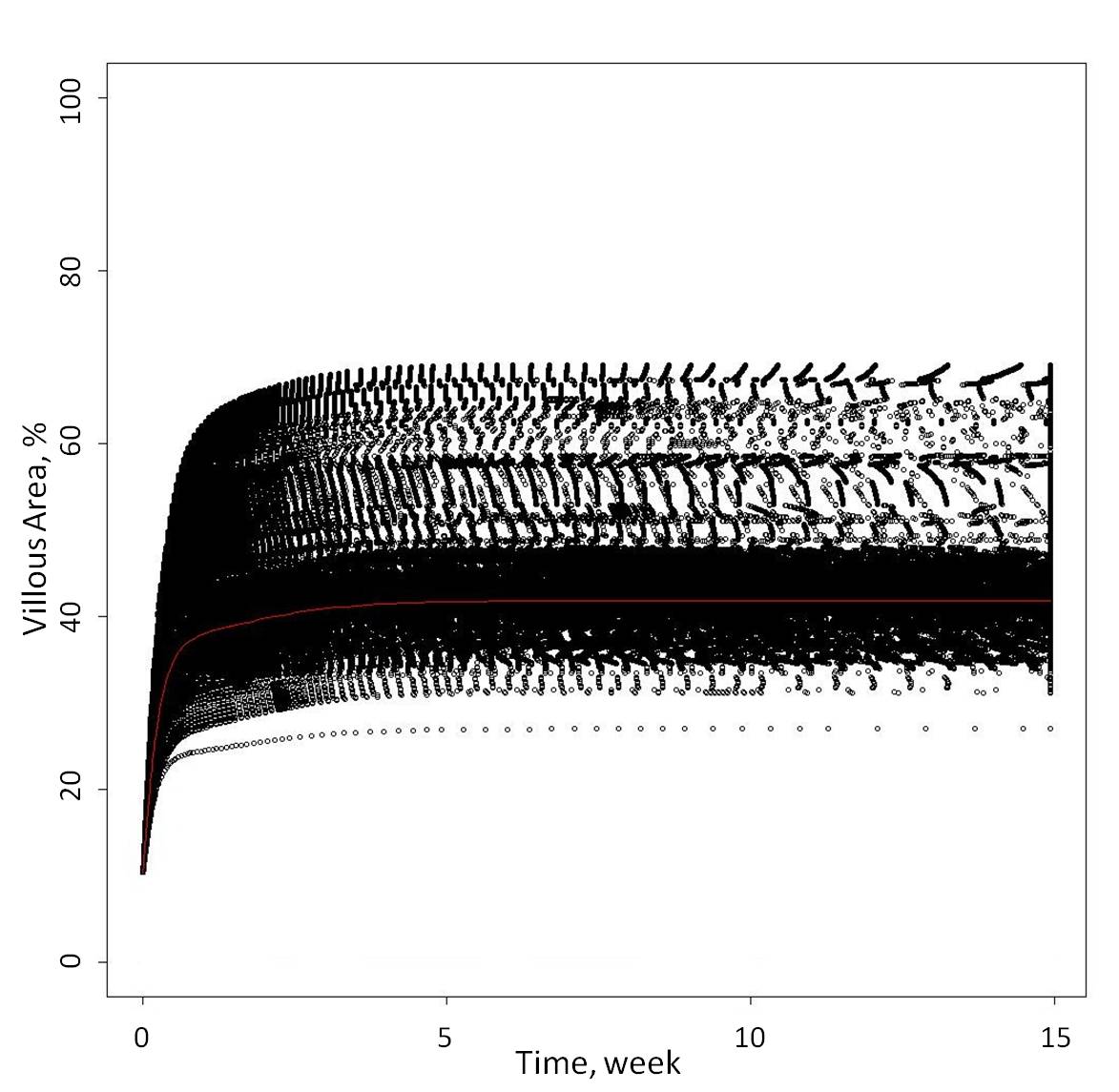


**Figure S23**

Simulation of IL-15 antibodies action on Villous Area drawing on the range of parameter’s confidential intervals. Red curve – simulation with optimum value of parameters. Points – simulations with parameters values obtained by Monte Carlo method from confidential intervals.


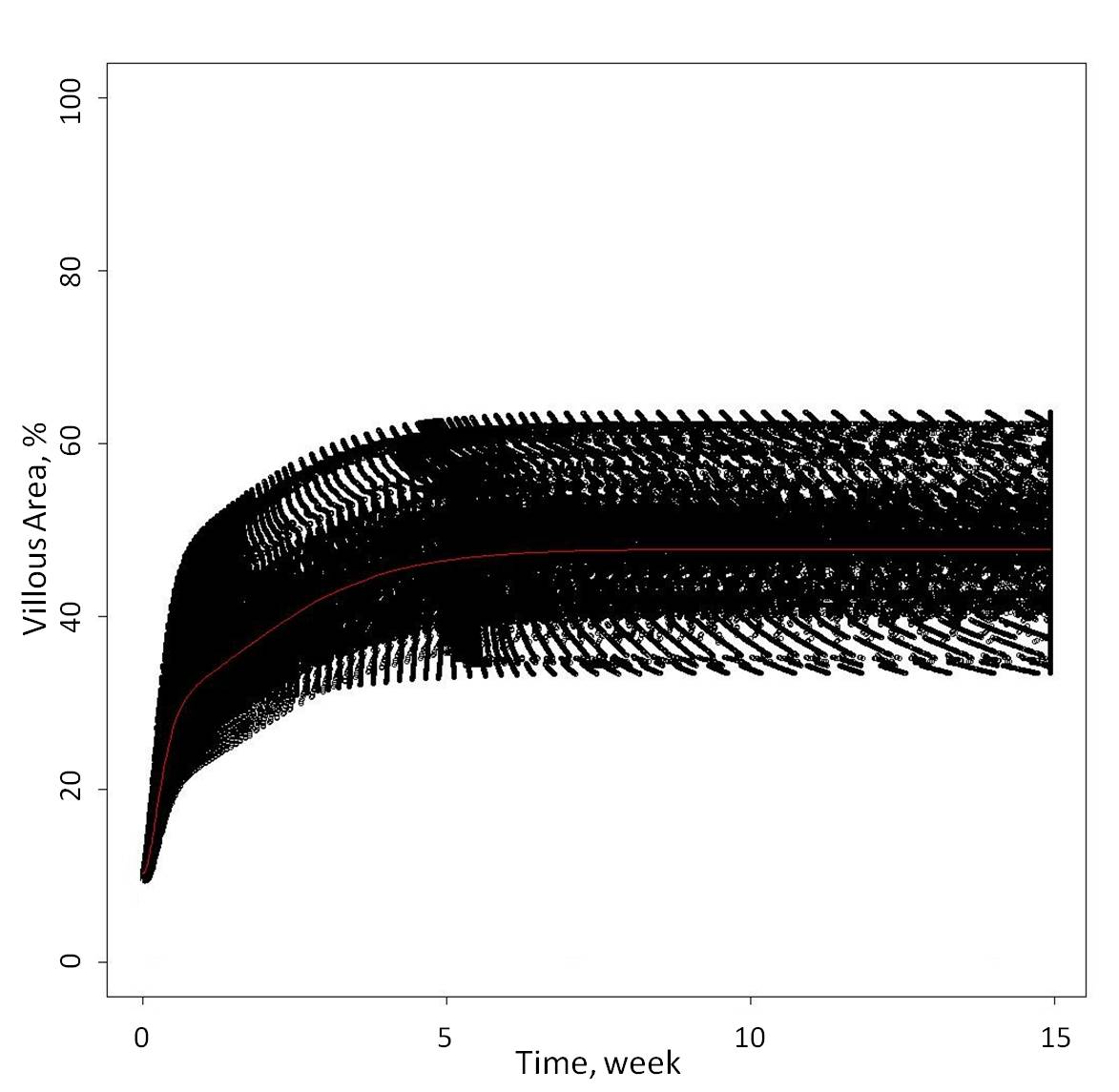


**Figure S24**

Simulation of TG-2 inhibitor action on Villous Area drawing on the range of parameter’s confidential intervals. Red curve – simulation with optimum value of parameters. Points – simulations with parameters values obtained by Monte Carlo method from confidential intervals.


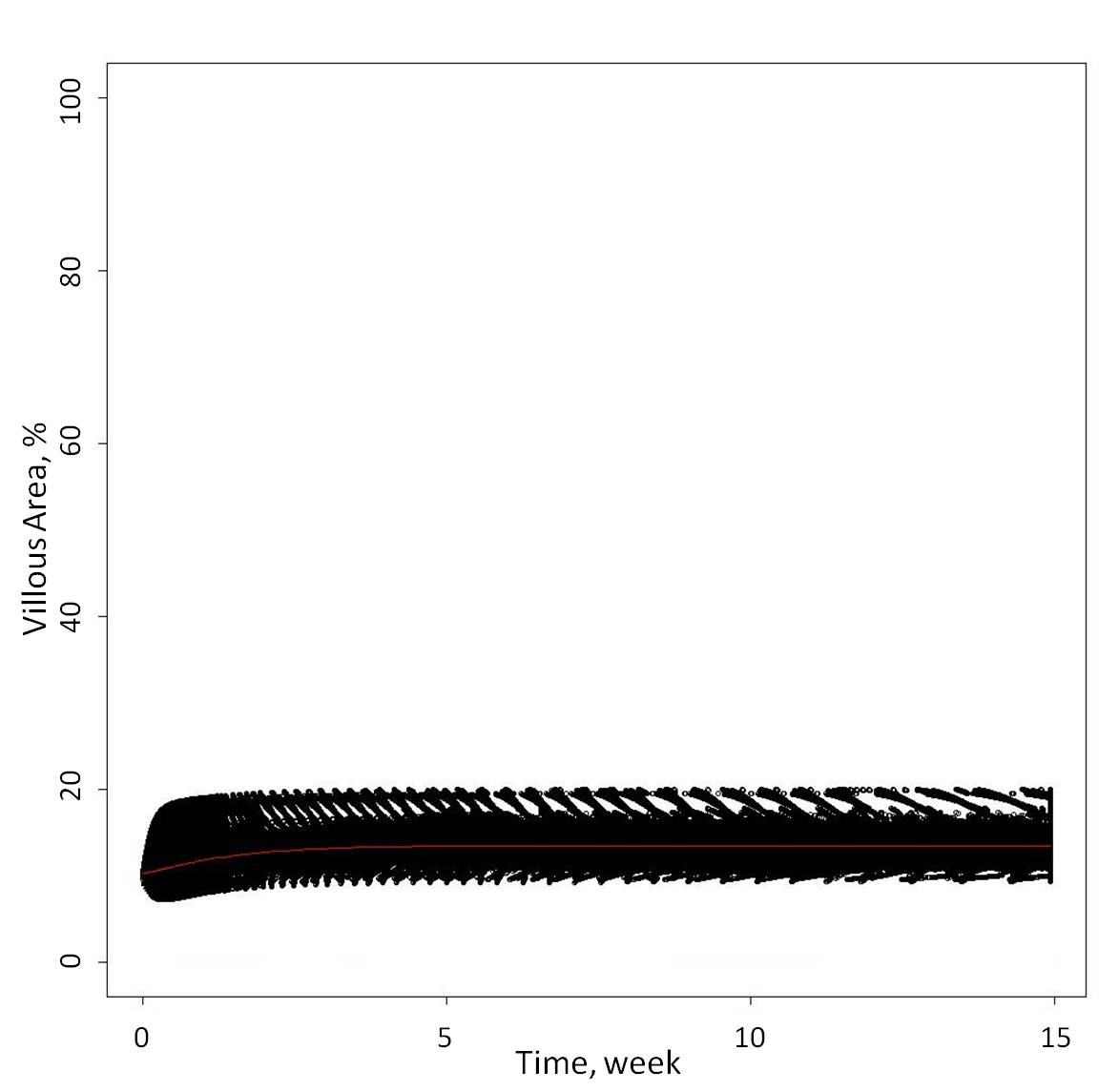


**Tables**

**Table 1**. List of variables.

| Variable | Description |
| --- | --- |
| $G_{gut}$ | Native gluten peptides in lumen |
| *IEC* | Nonactivated Intestinal Epithelial Cells |
| $IEC_{a}$ | Activated Intestinal Epithelial Cells |
| *IL15* | Interleukin-15 |
| *IEL* | Nonactivated Intraepithelial Lymphocytes |
| $IEL_{a}$ | Activated Intraepithelial Lymphocytes |
| $G_{muc}$ | Native gluten peptides in lamina |
| $G_{d}$ | Deamidated gluten peptides in lamina |
| *APC* | Nonactivated Antigen Presenting Cells |
| $APC_{a}$ | Activated Antigen Presenting Cells |
| *T* | Nonactivated T cells |
| $T_{i}$ | Intermediate T cells |
| $T_{a}$ | Activated T cells |
| *IF21* | Interferon Gamma + Interleukin-21 |
| *Ab* | Antibodies |
| *Zln* | Zonulin |

**Table 2**. List of parameters.

| Parameter | Reaction number | Value | 95% Confidential intervals | | Dimension | Method of verification |
| --- | --- | --- | --- | --- | --- | --- |
|  |  |  | Left value | Right value |  |  |
| $k_{\inf lux}$ | 1 | 750000**/**  7500 | - | - | pM/h | Average influx for different states (diets) |
| $k_{d}^{gut}$ | 2 | 0.26 | - | - | 1/h | Taken from the literature [15] |
| $k_{m}^{iec}$ | 3 | 0.0001315 | - | - | pM/h | Calculated against healthy patient steady state |
| $K_{d\_inh}^{iec}$ | 3 | 3490802 | 1986805 | 20940800 | pM | Fitted against GFD and CD steady state |
| $k_{d}^{iec}$ | 4,6 | 0.015462 | 0.015468 | 0.015474 | 1/h | Fitted against *in vitro* data [14] (Fig S9) |
| $K_{d}^{ab}$ | 4,6 | 3846978 | 1401749 | 99996980 | pM | Fitted against GFD and CD steady state |
| $K_{d}^{iel}$ | 4,6 | 0.003 | 0.0016 | 0.1553 | pM | Fitted against GFD and CD steady state |
| $K_{d}^{if21}$ | 4,6 | 43.39 | 31.82 | 66 | pM | Fitted against GFD and CD steady state |
| $k_{a}^{iec}$ | 5 | 0.5 | 0.4 | 0.64 | 1/h | Fitted against GFD and CD steady state |
| $K_{d}^{iec}$ | 5 | 5000 | - | - | pM | Taken from the literature [29] |
| *P* | 5 | 3 | - | - | - | Taken from the literature [30] |
| *N* | 5 | 10 | - | - | - | Taken from the literature [30] |
| $k_{s\_iec}^{il15}$ | 7 | 219 | 160 | 305 | 1/h | Fitted against GFD and CD steady state |
| $k_{s\_apc}^{il15}$ | 7 | 275 | 214 | 342 | 1/h | Fitted against GFD and CD steady state |
| $k_{d}^{il15}$ | 8 | 0.5794 | 0.5754 | 0.5834 | 1/h | Fitted against *in vitro* data [11] (Fig S5) |
| $k_{m}^{iel}$ | 9 | 0.00009 | - | - | pM/h | Calculated against healthy patient steady state |
| $k_{d}^{iel}$ | 10, 12 | 0.05492 | 0.05452 | 0.05533 | 1/h | Fitted against *in vitro* data [12] (Fig S6) |
| $K_{d\_inh}^{iel}$ | 10, 12 | 1152.5 | 971.5 | 1728.8 | pM | Fitted against *in vitro* data [12] (Fig S7) |
| $k_{a}^{iel}$ | 11 | 1.69 | 1.24 | 2.22 | 1/h | Fitted against GFD and CD steady state |
| $K_{d}^{1}$ | 11 | 11.11 | 8.26 | 15.4 | pM | Fitted against GFD and CD steady state |
| $k_{t}^{g}$ | 13 | 0.6411 | 0.5827 | 0.7063 | 1/h | Fitted against *ex vivo* data [15] (Fig S9) |
| $K_{d}^{z\ln}$ | 13 | 1.13 | 0.6 | 2.1 | pM | Fitted against *in vitro* data [19, 20. 21] (Fig S13) |
| $k^{z\ln}$ | 13 | 0.8 | 0.78 | 0.82 | - | Fitted against *in vitro* data [19, 20. 21] (Fig S13) |
| $k_{d}^{muc}$ | 14 | 0.26 | - | - | 1/h | Taken from the literature [15] |
| $k_{d}^{deam}$ | 15 | 0.26 | - | - | 1/h | Taken from the literature [15] |
| $k_{in}^{apc}$ | 16 | 0.0000096 | - | - | pM/h | Calculated against healthy patient steady state |
| $K_{d\_stim}^{il15}$ | 16 | 2.32 | 1.53 | 4.68 | pM | Fitted against GFD and CD steady state |
| $k_{d}^{apc}$ | 17, 19 | 0.0096 | - | - | 1/h | Taken from the literature [31] |
| $k_{a}^{apc}$ | 18 | 0.35 | 0.09 | 1.39 | 1/h | Fitted against GFD and CD steady state |
| $EC_{50}^{tg\_g}$ | 18, 25 | 700000 | - | - | pM | Taken from the literature [32, 33] |
| $EC_{50}^{deam}$ | 18, 25 | 700000 | - | - | pM | Taken from the literature [32, 33] |
| $EC_{50}^{muc}$ | 18, 25 | 3500000 | - | - | pM | Taken from the literature [32, 33] |
| $k_{m}^{t}$ | 20 | 0.0000058 | - | - | pM/h | Calculated against healthy patient steady state |
| $k_{d}^{t}$ | 21, 28 | 0.00675 | 0.006745 | 0.006798 | 1/h | Fitted against *in vitro* data [16] (Fig S11) |
| $k_{a}^{t}$ | 22 | 1.95 | 1.39 | 2.92 | 1/(pM*h) | Fitted against GFD and CD steady state |
| $K_{d\_stim}^{act}$ | 22 | 147 | 56 | 9958 | pM | Fitted against GFD and CD steady state |
| $k_{s\_1}^{if21}$ | 23 | 3303.1 | 3301.81 | 3304.39 | 1/h | Fitted against *in vitro* data [13] (Fig S8) |
| $k_{s\_2}^{if21}$ | 23 | 12071 | - | - | 1/h | Sum of IL-21 and IFN-γ (see below) |
| $k_{d}^{if21}$ | 24 | 0.15 | - | - | 1/h | Average between IL-21 and IFN-γ (see below) |
| $k_{s}^{ab}$ | 25 | 1.66 | 1.26 | 2.26 | pM/h | Fitted against GFD and CD steady state |
| $k_{ab}^{ta}$ | 25 | 114703 | 87142 | 156288 | - | Fitted against GFD and CD steady state |
| $K_{d\_ab}^{ta}$ | 25 | 0.0042 | 0.003 | 0.0056 | pM | Fitted against GFD and CD steady state |
| $k_{d}^{ab}$ | 26 | 0.001375 | - | - | 1/h | Taken from the literature [34] |
| $k_{ia}$ | 27 | 1.46 | 1.13 | 1.81 | 1/h | Fitted against GFD and CD steady state |
| $k_{d}^{ag\_ab}$ | 29 | 0.00811 | - | - | 1/h | Taken from the literature [35, 36] |
| $K_{d}^{ab\_2}$ | 29, 30 | 3573320 | 2479830 | 5675924 | pM | Fitted against GFD and CD steady state |
| $k_{rat}$ | 30 | 1.5 | - | - | 1/h | Taken from the literature [1] |
| $k_{s}^{z\ln}$ | 31 | 3327155 | 2606224 | 4612692 | 1/h | Fitted against GFD and CD steady state |
| $k_{d}^{z\ln}$ | 32 | 0.56 | 0.4 | 0.71 | 1/h | Fitted against GFD and CD steady state |
| $k_{d}^{cat}$ | 33 | 1140 | - | - | 1/h | Taken from the literature [37] |
| *e0* | 33 | 35087.72 | - | - | pM | Taken from the literature [38] |
| $K_{d\_ab}^{e}$ | 33 | 147292.3 | 144390.1 | 150264.7 | pM | Fitted against *in vitro* data [17, 18] (Fig S12) |
| $K_{m}^{deam}$ | 33 | 250000000 | - | - | pM | Taken from the literature [37] |
| $k_{a}^{i}$ | 34 | 0.01 | - | - | 1/h | Assumed |
| $k_{va}$ | VA function | 48.23 | 24.72 | 147.71 | - | Fitted against *in vivo* data [28] (Fig S14) |
| Parameters for IFN gamma | | | | | | |
| $k_{d}^{if21}$ | 24 | 0.1 | - | - | 1/h | Taken from the literature [39] |
| $k_{s\_2}^{if21}$ | 23 | 9256.74 | 9256.63 | 9256.85 | 1/h | Fitted against *in vitro* data [9] (Fig S3) |
| Parameters for IL-21 | | | | | | |
| $k_{d}^{if21}$ | 24 | 0.231 | - | - | 1/h | Taken from the literature [40] |
| $k_{s\_2}^{if21}$ | 23 | 2814.39 | 2814.32 | 2814.46 | 1/h | Fitted against *in vitro* data [10] (Fig S4) |

**Table 3**. Data on healthy subjects

| Variable | Value, pM | Reference |
| --- | --- | --- |
| *IEC* | 0.0085 | [26, 27] |
| *IEL* | 0.0017 | [25] |
| *APC* | 0.001 | [24] |
| *T* | 0.00085 | [25] |

**Table 4**. Quality of fitting against *in vivo* steady state data.

| Variable | Gluten-containing diet | | Gluten free diet | | Reference |
| --- | --- | --- | --- | --- | --- |
|  | Experimental value | Model value | Experimental value | Model value |  |
| *Ab* | 1 µM | 0.99 µM | - | - | [22] |
| *Zln* | 2 nM | 1.998 nM | - | - | [23] |
| *IL15* | 0.8 pM | 0.821 pM | 0.56 pM | 0.56 pM | [13] |
| *IEC* | 0.0018 pM | 0.00181 pM | - | - | [26, 27] |
| $IEC_{a}$ | 0.0006 pM | 0.00061 pM | - | - | [26, 27] |
| *Total IEC* | 0.0024 pM | 0.00242 pM | 0.00425 | 0.00429 | [26, 27] |
| *IEL* | 0.0006 pM | 0.00054 pM | - | - | [25] |
| $IEL_{a}$ | 0.0014 pM | 0.00116 pM | - | - | [25] |
| *Total IEL* | 0.002 pM | 0.0017 pM | 0.0017 | 0.0017 | [25] |
| *Total APC* | 0.0015 pM | 0.00136 pM | 0.001 | 0.00125 | [24] |
| *Total T cells* | 0.001 pM | 0.00103 pM | 0.00095 | 0.00089 | [25] |

**References**

1. Aleanzi M, Demonte AM, Esper C, Garcilazo S, Waggener M: **Celiac disease: antibody recognition against native and selectively deamidated gliadin peptides.** *Clin Chem* 2001, **47**:2023-2028.

2. Cornish-Bowden A: *Fundamentals of enzyme kinetics (3rd ed.).* London: Portland Press; 2004.

3. Demin O, Goryanin I: *Kinetic Modelling in Systems Biology.* London: CRC Press; 2008.

4. Thomson AB, Drozdowski L, Iordache C, Thomson BK, Vermeire S, Clandinin MT, Wild G: **Small bowel review: Normal physiology, part 1.** *Dig Dis Sci* 2003, **48**:1546-1564.

5. Thomson AB, Drozdowski L, Iordache C, Thomson BK, Vermeire S, Clandinin MT, Wild G: **Small** **bowel review: Normal physiology, part 2.** *Dig Dis Sci* 2003, **48**:1565-1581.

6. Holt PR: **The small intestine.** *Clin Gastroenterol* 1985, **14**:689-723.

7. Hasan M, Ferguson A: **Measurements of intestinal villi non-specific and ulcer-associated duodenitis-correlation between area of microdissected villus and villus epithelial cell count.** *J Clin Pathol* 1981, **34**:1181-1186.

8. Guix M, Skinner JM, Whitehead R: **Measuring intraepithelial lymphocytes, surface area, and volume of lamina propria in the jejunal mucosa of coeliac patients.** *Gut* 1979, **20**:275-278.

9. Camarca A, Anderson RP, Mamone G, Fierro O, Facchiano A, Costantini S, Zanzi D, Sidney J, Auricchio S, Sette A, Troncone R, Gianfrani C: **Intestinal T cell responses to gluten peptides are largely heterogeneous: implications for a peptide-based therapy in celiac disease.** *J Immunol* 2009, **182**:4158-4166.

10. Schmitt N, Morita R, Bourdery L, Bentebibel SE, Zurawski SM, Banchereau J, Ueno H: **Human dendritic cells induce the differentiation of interleukin-21-producing T follicular helper-like cells through interleukin-12.** *Immunity* 2009, **31**:158-169.

11. Bergamaschi C, Rosati M, Jalah R, Valentin A, Kulkarni V, Alicea C, Zhang GM, Patel V, Felber BK, Pavlakis GN: **Intracellular interaction of interleukin-15 with its receptor alpha during production leads to mutual stabilization and increased bioactivity.** *J Biol Chem* 2008, **283**:4189-99.

12. Lai YG, Hou MS, Hsu YW, Chang CL, Liou YH, Tsai MH, Lee F, Liao NS: **IL-15 does not affect IEL development in the thymus but regulates homeostasis of putative precursors and mature CD8 alpha alpha+ IELs in the intestine.** *J Immunol* 2008, **180**:3757-3765.

13. Di Sabatino A, Ciccocioppo R, Cupelli F, Cinque B, Millimaggi D, Clarkson MM, Paulli M, Cifone MG, Corazza GR: **Epithelium derived interleukin 15 regulates intraepithelial lymphocyte Th1 cytokine production, cytotoxicity, and survival in coeliac disease.** *Gut* 2006, **55**:469-477.

14. Di Sabatino A, Ciccocioppo R, D'Alo S, Parroni R, Millimaggi D, Cifone MG, Corazza GR: **Intraepithelial and lamina propria lymphocytes show distinct patterns of apoptosis whereas both populations are active in Fas based cytotoxicity in coeliac disease.** *Gut* 2001, **49**:380-386.

15. Matysiak-Budnik T, Candalh C, Dugave C, Namane A, Cellier C, Cerf-Bensussan N, Heyman M: **Alterations of the intestinal transport and processing of gliadin peptides in celiac disease.** *Gastroenterology* 2003, **125**:696-707.

16. Di Sabatino A, D'Alo S, Millimaggi D, Ciccocioppo R, Parroni R, Sciarra G, Cifone MG, Corazza GR: **Apoptosis and peripheral blood lymphocyte depletion in coeliac disease.** *Immunology* 2001, **103**:435-440.

17. Byrne G, Feighery C, Jackson J, Kelly J: **Coeliac disease autoantibodies mediate significant inhibition of tissue transglutaminase.** *Clin Immunol* 2010, **136**:426-431.

18. Dieterich W, Trapp D, Esslinger B, Leidenberger M, Piper J, Hahn E, Schuppan D: **Autoantibodies of patients with coeliac disease are insufficient to block tissue transglutaminase activity.** *Gut* 2003, **52**:1562-1566.

19. Tripathi A, Lammers KM, Goldblum S, Shea-Donohue T, Netzel-Arnett S, Buzza MS, Antalis TM, Vogel SN, Zhao A, Yang S, Arrietta MC, Meddings JB, Fasano A: **Identification of human zonulin, a physiological modulator of tight junctions, as prehaptoglobin-2.** *Proc Natl Acad Sci U S A* 2009, **106**:16799-16804.

20. El Asmar R, Panigrahi P, Bamford P, Berti I, Not T, Coppa GV, Catassi C, Fasano A: **Host-dependent zonulin secretion causes the impairment of the small intestine barrier function after bacterial exposure.** *Gastroenterology* 2002, **123**:1607-1615.

21. Wang W, Uzzau S, Goldblum SE, Fasano A: **Human zonulin, a potential modulator of intestinal tight junctions.** *J Cell Sci* 2000, **113**:4435-4440.

22. Wood GM, Shires S, Howdle PD, Losowsky MS: **Immunoglobulin production by coeliac biopsies in organ culture.** *Gut* 1986, **27**:1151-1160.

23. Sapone A, de Magistris L, Pietzak M, Clemente MG, Tripathi A, Cucca F, Lampis R, Kryszak D, Carteni M, Generoso M, Iafusco D, Prisco F, Laghi F, Riegler G, Carratu R, Counts D, Fasano A: **Zonulin upregulation is associated with increased gut permeability in subjects with type 1 diabetes and their relatives.** *Diabetes* 2006, **55**:1443-1449.

24. Raki M, Tollefsen S, Molberg O, Lundin KE, Sollid LM, Jahnsen FL: **A unique dendritic cell subset accumulates in the celiac lesion and efficiently activates gluten-reactive T cells.** *Gastroenterology* 2006, **131**:428-438.

25. Verkasalo MA, Arato A, Savilahti E, Tainio VM: **Effect of diet and age on jejunal and circulating lymphocyte subsets in children with coeliac disease: persistence of CD4-8-intraepithelial T cells through treatment.** *Gut* 1990, **31**:422-425.

26. Ferguson A, Murray D: **Quantitation of intraepithelial lymphocytes in human jejunum.** *Gut* 1971, **12**:988-994

27. Mazzarella G, Maglio M, Paparo F, Nardone G, Stefanile R, Greco L, van de Wal Y, Kooy Y, Koning F, Auricchio S, Troncone R: **An immunodominant DQ8 restricted gliadin peptide activates small intestinal immune response in in vitro cultured mucosa from HLA-DQ8 positive but not HLA-DQ8 negative coeliac patients.** *Gut* 2003, **52**:57-62.

28. Cummins AG, Thompson FM, Butler RN, Cassidy JC, Gillis D, Lorenzetti M, Southcott EK, Wilson PC: **Improvement in intestinal permeability precedes morphometric recovery of the small intestine in coeliac disease.** *Clin Sci (Lond)* 2001, **100**:379-386.

29. Neote K, Letts LG, Moser B: *Chemokine Biology - Basic Research and Clinical Application.*  Basel: Birkhauser Basel; 2007.

30. Loetscher M, Loetscher P, Brass N, Meese E, Moser B: **Lymphocyte-specific chemokine receptor CXCR3: regulation, chemokine binding and gene localization.** *Eur J Immunol* 1998, **28**:3696-3705.

31. Kamath AT, Pooley J, O'Keeffe MA, Vremec D, Zhan Y, Lew AM, D'Amico A, Wu L, Tough DF, Shortman K: **The development, maturation, and turnover rate of mouse spleen dendritic cell populations.** *J Immunol* 2000, **165**:6762-6770.

32. Shan L, Molberg O, Parrot I, Hausch F, Filiz F, Gray GM, Sollid LM, Khosla C: **Structural basis for gluten intolerance in celiac sprue.** *Science* 2002, **297**:2275-2279.

33. Gianfrani C, Siciliano RA, Facchiano AM, Camarca A, Mazzeo MF, Costantini S, Salvati VM, Maurano F, Mazzarella G, Iaquinto G, Bergamo P, Rossi M: **Transamidation of Wheat Flour Inhibits the Response to Gliadin of Intestinal T Cells in Celiac Disease.** *Gastroenterology* 2007, **133**:780-789.

34. Coico R, Sunshine G: *Immunobiology: A Short Course (6th edition).* New Jersey: Hoboken; 2009.

35. Davis CB, Tobia LP, Kwok DC, Oishi CM, Kheterpal N, Hepburn TW, Benincosa LJ, Chow FS, Jusko WJ: **Accumulation of Antibody-Target Complexes and the Pharmacodynamics of Clotting after Single Intravenous Administration of Humanized Anti-Factor IX Monoclonal Antibody to Rats.** *Drug Delivery* 1999, **6**:171–179.

36. Benincosa LJ, Chow FS, Tobia LP, Kwok DC, Davis CB, Jusko WJ: **Pharmacokinetics and pharmacodynamics of a humanized monoclonal antibody to factor IX in cynomolgus monkeys.** *J Pharmacol Exp Ther* 2000, **292**:810-816.

37. Pinkas DM, Strop P, Brunger AT, Khosla C: **Transglutaminase 2 undergoes a large conformational change upon activation.** *PLoS Biol* 2007, **5**:e327.

38. Skovbjerg H, Koch C, Anthonsen D, Sjostrom H: **Deamidation and cross-linking of gliadin peptides by transglutaminases and the relation to celiac disease.** *Biochim Biophys Acta* 2004, **1690**:220-230.

39. [http://www.anticancer.net/resan/gamma_interferon.php]

40. Thompson JA, Curti BD, Redman BG, Bhatia S, Weber JS, Agarwala SS, Sievers EL, Hughes SD, DeVries TA, Hausman DF: **Phase I study of recombinant interleukin-21 in patients with metastatic melanoma and renal cell carcinoma.** *J Clin Oncol* 2008, **26**:2034-2039.
